# *SETD8*, a frequently mutated gene in cervical cancer, enhances cisplatin sensitivity by impairing DNA repair

**DOI:** 10.1186/s13578-023-01054-y

**Published:** 2023-06-12

**Authors:** Xin Wang, Chen Cao, Xiangyu Tan, Xueyao Liao, Xiaofang Du, Xueqian Wang, Ting Liu, Danni Gong, Zheng Hu, Xun Tian

**Affiliations:** 1 Department of Obstetrics and Gynecology, Academician Expert Workstation, The Central Hospital of Wuhan, Tongji Medical College, Huazhong University of Science and Technology, Wuhan, Hubei 430014 China; 2 Department of Gynecological Oncology, Tongji Hospital, Tongji Medical College, Huazhong University of Science and Technology, Wuhan, Hubei 430030 China; 3 Department of Gynecologic Oncology, Women and Children’s Hospital Affiliated to Zhongnan Hospital of Wuhan University, Wuhan, Hubei 430071 China; 4 Department of Radiation and Medical Oncology, Zhongnan Hospital of Wuhan University, Wuhan, Hubei 430071 China; 5 Hubei Key Laboratory of Tumor Biological Behaviors, Zhongnan Hospital of Wuhan University, Wuhan, Hubei 430071 China; 6 Hubei Cancer Clinical Study Center, Zhongnan Hospital of Wuhan University, Wuhan, Hubei 430071 China; 7 National Clinical Research Center for Obstetrics and Gynecology, Cancer Biology Research Center (Key Laboratory of the Ministry of Education), Tongji Hospital, Tongji Medical College, Huazhong University of Science and Technology, Wuhan, Hubei 430030 China

**Keywords:** Cisplatin sensitivity, *SETD8*, Cervical cancer, DNA repair, Whole exome sequencing

## Abstract

**Background:**

Cisplatin is commonly used to treat cervical cancer while drug resistance limits its effectiveness. There is an urgent need to identify strategies that increase cisplatin sensitivity and improve the outcomes of chemotherapy.

**Results:**

We performed whole exome sequencing (WES) of 156 cervical cancer tissues to assess genomic features related to platinum-based chemoresistance. By using WES, we identified a frequently mutated locus *SETD8* (7%), which was associated with drug sensitivity. Cell functional assays, in vivo xenografts tumor growth experiments, and survival analysis were used to investigate the functional significance and mechanism of chemosensitization after *SETD8* downregulation. Knockdown of *SETD8* increased the responsiveness of cervical cancer cells to cisplatin treatment. The mechanism is exerted by reduced binding of 53BP1 to DNA breaks and inhibition of the non-homologous end joining (NHEJ) repair pathway. In addition, *SETD8* expression was positively correlated with resistance to cisplatin and negatively associated with the prognosis of cervical cancer patients. Further, UNC0379 as a small molecule inhibitor of *SETD8* was found to enhance cisplatin sensitivity both in vitro and in vivo.

**Conclusions:**

*SETD8* was a promising therapeutic target to ameliorate cisplatin resistance and improve the efficacy of chemotherapy.

**Supplementary Information:**

The online version contains supplementary material available at 10.1186/s13578-023-01054-y.

## Background

Cervical cancer constitutes one of the most common cancers of the female reproductive system. Each year, approximately 570,000 women worldwide are diagnosed while more than 311,000 die from the disease [1]. Platinum-based chemotherapy is a standard treatment for cervical cancer [2]. Cisplatin acts to destroy the template function of the DNA double helix by forming intra-chain and inter-chain adducts, leading to DNA damage [3]. By this mechanism, cisplatin inhibits replication and transcription of DNA, resulting in apoptosis of cancer cell [4]. However, the remission rate of recurrent and advanced cervical cancer due to chemotherapy is only approximately 25%, with mean patient survival time being less than 1 year [5]. One confounding factor which compromises treatment success is the resistance to platinum-based drugs [6]. These observations indicate the need to investigate mechanisms of drug resistance. Such studies are likely to have important translational impacts on the survival of patients with advanced cancers.

Various mechanisms are possible for the development of resistance to platinum-based drugs. These include increased DNA repair [7], reduced platinum uptake [8], increased platinum efflux [9], increased platinum inactivation [10] and inhibition of the apoptosis pathway [11]. Among them, increased rate of DNA repair is considered as a key factor for cisplatin resistance. Many thousands of DNA mutations are involved which have contributed to the evolution of the cancer genome [12]. Therefore, whole exome sequencing (WES) has been used to identify driver gene mutations related to tumor resistance[13, 14]. Identification of such mutations provides targets for overcoming tumor resistance. For instance, WES was applied to identify the ERCC2 mutations which are related to cisplatin sensitivity in bladder cancer[15] and CCNE1 mutations which are related to cisplatin resistance in ovarian cancer[16].

*SETD8* (also known as *PR-Set7*, *SET8*, or *KMT5A*) encodes a lysine methyltransferase-containing SET domain, which is the only activity responsible for the mono-methylation of H4K20 (H4K20me1) [17]. H4K20 methylation plays a key role in DNA replication, DNA strand break repair and heterochromatin silencing [18, 19]. In addition to H4K20, SETD8 protein is able to perform lysine-methylations of many other cancer-related proteins. For example, *SETD8* protein performs mono-methylation of p53 (p53K382me1) on lysine 382 which inhibits p53-dependent transcriptional activation in tumor cells [20]. Moreover, SETD8 protein may further regulate the p53-mediated apoptosis pathway by methylating Numb [21]. SETD8 protein is able to mono-methylate *PCNA* on lysine 248 (*PCNAK248me1*) and stabilize PCNA protein by inhibiting poly-ubiquitination and enhancing the interaction between PCNA and FEN1 [22]. *SETD8* has been found to be overexpressed in various tumor types and to be involved in the process of cancer progression and metastasis [22–24].

In the current investigation, we used WES to profile genomic features related to the response of cervical cancer to platinum-based chemotherapy. A frequently mutated gene *SETD8* was identified as being closely related to cisplatin-sensitivity in patients. We found that *SETD8* mutations played an important role in the process of DNA repair, contributing to the cisplatin-sensitive response to DNA damage. Furthermore, we showed that the small molecule inhibitor of *SETD8*, UNC0379, enhances the efficacy of cisplatin in vitro and in vivo.

## Results

### Mutations in ***SETD8*** influence responsiveness to platinum-based chemotherapy in cervical cancer and inhibition of *SETD8* enhances cisplatin sensitivity

**Fig. 1:**
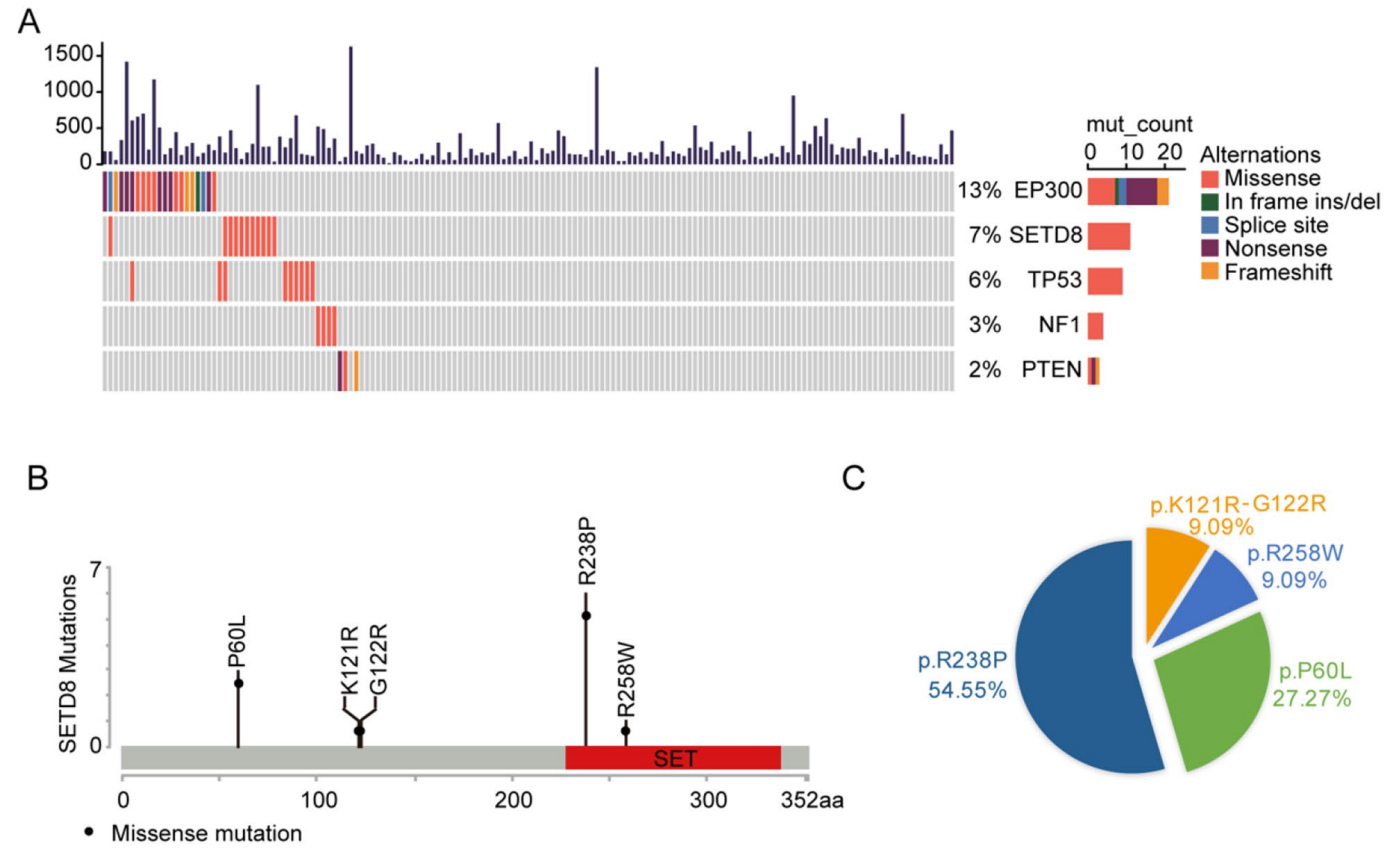
Landscapes of *SETD8* mutation in cervical cancer. (**A**) Mutation distribution and frequency of *SETD8* and other known high-frequency mutant genes in 156 cervical cancer patients. (**B**) Mutation form and its position in the *SETD8* sequence. (**C**) Frequencies of different mutated forms of *SETD8*.

Whole exome sequencing was conducted on tumor tissues from 156 cervical cancer patients prior to chemotherapy (supplementary Table S1). Well-known driver mutations were identified, including *EP300* (13%), *TP53* (6%), *NF1* (3%) and *PTEN* (2%) (Fig. 1A). Mutation frequencies were similar to those previously reported [25, 26]. Additionally, we identified a novel mutated gene *SETD8* with a frequency of 7% (Fig. 1A). *SETD8* mutations were distributed throughout the coding sequence of the gene with a total of 5 missense mutations (P60L, K121R, G122R, R238P and R258W) (supplementary Table S2). Two missense mutations, R238P and R258W, were located in the SET domain of *SETD8* (Fig. 1B). R238P was the most frequently mutated form of *SETD8* (54.55%), followed by P60L (27.27%), K121R&G122R (9.09%) and R258W (9.09%) (Fig. 1C). The functional impacts of the above mutations were analyzed (supplementary Table S3). The tumor mutational burden (TMB) was calculated [27] and the mean was found to be 8.26 mut/Mb for the cohort. TMB data for each tumor was presented in supplementary Table S4. We compared the TMB in *SETD8* mutated tumors with those of other tumors and found that *SETD8* mutations did not show statistically significant correlation with tumors with higher TMB (*p* = 0.6562, unpaired Student’s *t* test) (supplementary Fig. S1A).

**Fig. 2:**
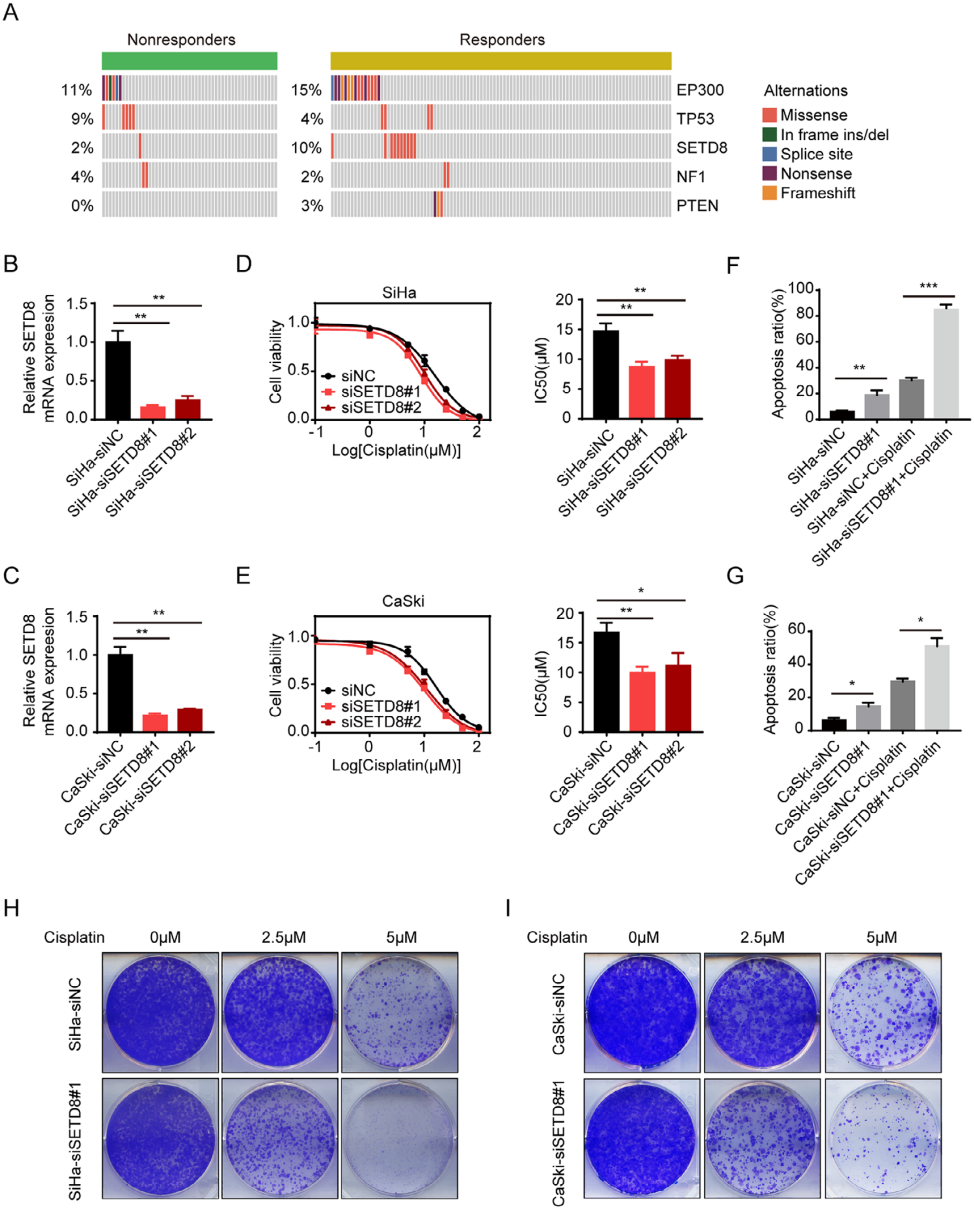
Targeting *SETD8* sensitizes cisplatin treatmentin vitro. (**A**) Distribution of *SETD8* and other high-frequency mutant genes in chemotherapy responders and non-responders. (**B**) Knockdown efficiency of *SETD8* siRNAs in SiHa cells. **: *p* < 0.01. (**C**) Knockdown efficiency of *SETD8* siRNAs in CaSki cells. (**D**) Dose-response curves of SiHa cells with cisplatin after transfection with two different si*SETD8* compared to siNC. IC50 values were derived from the dose-response assay indicating that responsiveness to cisplatin is significantly increased in SiHa after transfection with si*SETD8*#1 and si*SETD8*#2. (**E**) Dose-response curves of CaSki cells to cisplatin after transfection with two different si*SETD8* compared to siNC. IC50 values were derived from the dose-response assay. (**F**) SiHa cells transfected with siNC and siSETD8#1 were treated with 20 µM for 48 h. The apoptotic of SiHa cells were assayed by Annexin V-FITC/PI staining. (**G**) CaSki cells transfected with siNC and siSETD8#1 were treated with 20 µM for 48 h. The apoptotic of CaSki cells were assayed by Annexin V-FITC/PI staining. Error bars represent ± SD from three replicates. *p* values were determined by two-tailed Student’s *t *test (ns: not significant; *: 0.01 ≤ *p* < 0.05; **: 0.001 ≤ *p* < 0.01; ***: 0.0001 ≤ *p* < 0.001; ****: *p* < 0.0001). (**H-I**) Colony formation assays were performed using SiHa and CaSki cells with *SETD8* knockdown and cisplatin treatment. *p* values were determined by two-tailed Student’s *t* test and Fisher’s exact test (ns: not significant; *: 0.01 ≤ *p* < 0.05; **: 0.001 ≤ *p* < 0.01; ***: 0.0001 ≤ *p* < 0.001; ****: *p* < 0.0001)

All 156 cervical cancer patients received platinum-based neoadjuvant chemotherapy (NACT). The patients were divided into responders (n = 104) and non-responders’ group (n = 52) according to the results of the chemotherapy. The mutation frequency of *SETD8* was higher (10%) among the cisplatin responders (Fig. 2A). To further study the relationship between *SETD8* and drug response, we used 2 different siRNAs to knockdown *SETD8* expression in two cultured cell-lines derived from cervical cancer cells, SiHa and CaSki cells, confirming its depletion by qPCR (Fig. 2B C, respectively). The knockdown efficiencies of si*SETD8*#1 and si*SETD8*#2 were both more than 70% in both SiHa and CaSki cells. Dose-response curves of SiHa cells (Fig. 2D) and CaSki cells (Fig. 2E) to cisplatin were compared with a negative control siNC. After knockdown of *SETD8*, the sensitivity to cisplatin was increased as demonstrated by decreased IC50 in both SiHa cells (si*SETD8*#1: 14.71 ± 0.75 µM to 8.73 ± 0.49 µM, *p* = 0.0027; si*SETD8*#2: 14.71 ± 0.75 µM to 9.88 ± 0.40 µM, *p* = 0.0049) and CaSki cells (si*SETD8*#1: 16.69 ± 0.97 µM to 9.97 ± 0.58 µM, *p* = 0.0040; si*SETD8*#2: 16.69 ± 0.97 µM to 11.17 ± 1.12 µM, *p* = 0.0239) (Fig. 2D, E). Flow cytometry indicated increased cisplatin-induced apoptosis after knockdown of *SETD8* in both SiHa cells (30.24% ± 2.00% to 84.92 ± 2.96%; *p* = 0.0001) and CaSki cells (29.61% ± 1.91–51.04% ± 4.84%; *p* = 0.0146) (Fig. 2F, G). Knockdown of *SETD8* also reduced colony formation by SiHa and CaSki cells at various cisplatin concentrations (Fig. 2H-I). These results demonstrated that *SETD8* inhibition enhances the sensitivity of cervical cancer cells to cisplatin.

### Down-regulation of *SETD8* enhances cisplatin sensitivity by reducing the methylation of H4K20 and inhibiting the NHEJ DNA repair pathway

**Fig. 3:**
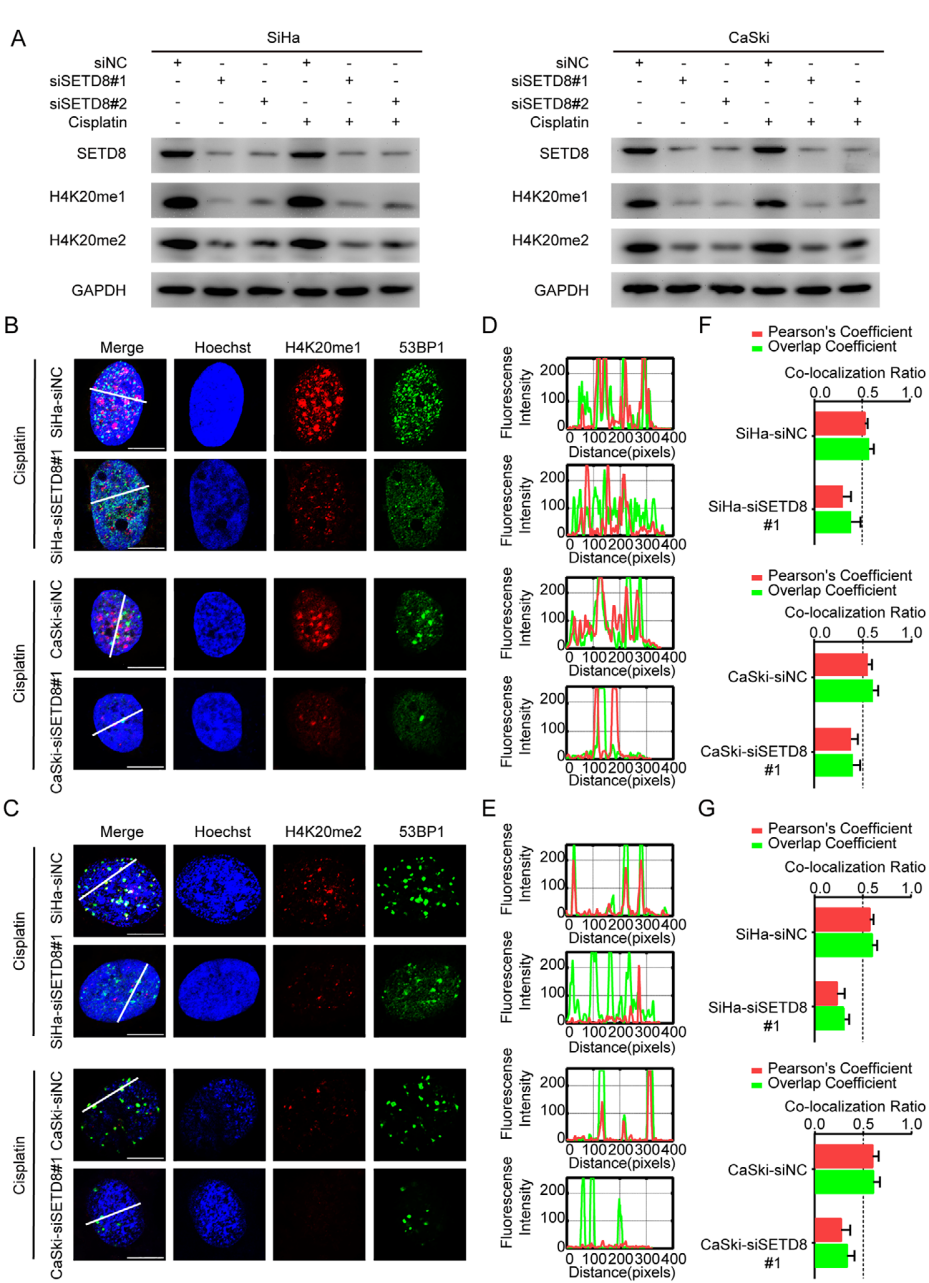
*SETD8* inhibition affects 53BP1 accumulation by reducing the methylation level of H4K20. (**A**) Western blot analysis of SETD8 and H4K20me1/H4K20me2 levels. Cells transfected with two different SETD8 siRNAs were treated with cisplatin for 24 h compared to siNC. (**B**) Immunofluorescence for H4K20me1 and 53BP1 post cisplatin in SiHa and CaSki cells, Scale bar, 10 μm. (**C**) Immunofluorescence for H4K20me2 and 53BP1 post cisplatin in SiHa and CaSki cells, Scale bar, 10 μm. (**D-E**) Plot profiles show fluorescence intensity along an oblique line quantified by ImageJ. (F-G) Pearson’s Coefficient and Overlap Coefficient calculated with JACoP by ImageJ. Pearson’s Coefficient or Overlap Coefficient > 0.5 indicates co-localization

*SETD8* encodes the only H4K20 (H4K20me1) methyltransferase, which can further methylate H4K20me1 to H4K20me2 [17]. The lack of *SETD8* expression resulted not only in depletion of H4K20me1 but also in reduced H4K20me2 levels [28]. To further illuminate the situation, we assessed H4K20me1/2 levels in the presence of si*SETD8*. Western blots showed that *SETD8* knockdown reduced levels of H4K20me1 (mono-methylated) and H4K20me2 (di-methylated) in both cisplatin-treated and control cells (Fig. 3A). Reduced methylation of H4K20 were observed both in SiHa and CaSki cells and the effect of reduced H4K20 methylation levels needs further study (Fig. 3A, B).

The presence of histone H4 methylated on lysine 20 (H4K20me) is considered to be necessary for the recruitment of 53BP1 to double strand breaks (DSBs) [29]. Thus, we hypothesized that depletion of *SETD8* activity may reduce the binding of 53BP1 to DNA strand breaks via down-regulation of H4K20 methylation. Immunofluorescence experiments confirmed that reduced H4K20me1/2 levels due to *SETD8* knockdown impact binding of 53BP1 to DNA strand breaks in SiHa and CaSki cells with cisplatin treatment (Fig. 3B, C; supplementary Fig. S2A-B). To validate co-localization of H4K20me1 and H4K20me2 foci with 53BP1 foci, we used quantified fluorescence intensity (imageJ) to generate plot profiles and illustrate the degree of overlap (Fig. 3D, E). Enhanced accuracy was provided by use of imageJ co-localization Plugin, JACoP [30], to calculate the co-localization ratio (Pearson’s Coefficient and Overlap Coefficient ratio > 0.5 as criteria, Fig. 3F, G). Our analysis clearly showed extensive co-localization of H4K20me1and H4K20me2 foci with 53BP1 foci post cisplatin treatment. However, following *SETD8* knockdown, H4K20me1 and H4K20me2 signals decreased and did not co-localize with 53BP1 (Fig. 3B, C). Decreased binding of 53BP1 to DNA strand breaks in *SETD8* knockdown cells was further verified by quantitative analysis of fluorescence intensity plot profiles (Fig. 3D, E) and the co-localization ratio in SiHa cells (Pearson’s Coefficient: H4K20me1, 0.53 ± 0.03 to 0.30 ± 0.09; H4K20me2, 0.57 ± 0.04 to 0.24 ± 0.08; Overlap Coefficient: H4K20me1, 0.57 ± 0.05 to 0.38 ± 0.10; H4K20me2, 0.60 ± 0.05 to 0.31 ± 0.05). Similar results were obtained with CaSki cells (Pearson’s Coefficient: H4K20me1, 0.55 ± 0.04 to 0.38 ± 0.07; H4K20me2, 0.60 ± 0.06 to 0.27 ± 0.09; Overlap Coefficient: H4K20me1, 0.60 ± 0.06 to 0.40 ± 0.08; H4K20me2, 0.60 ± 0.07 to 0.33 ± 0.08) (Fig. 3F, G). In summary, we found that H4K20me1/2 was co-localized with 53BP1.

**Fig. 4:**
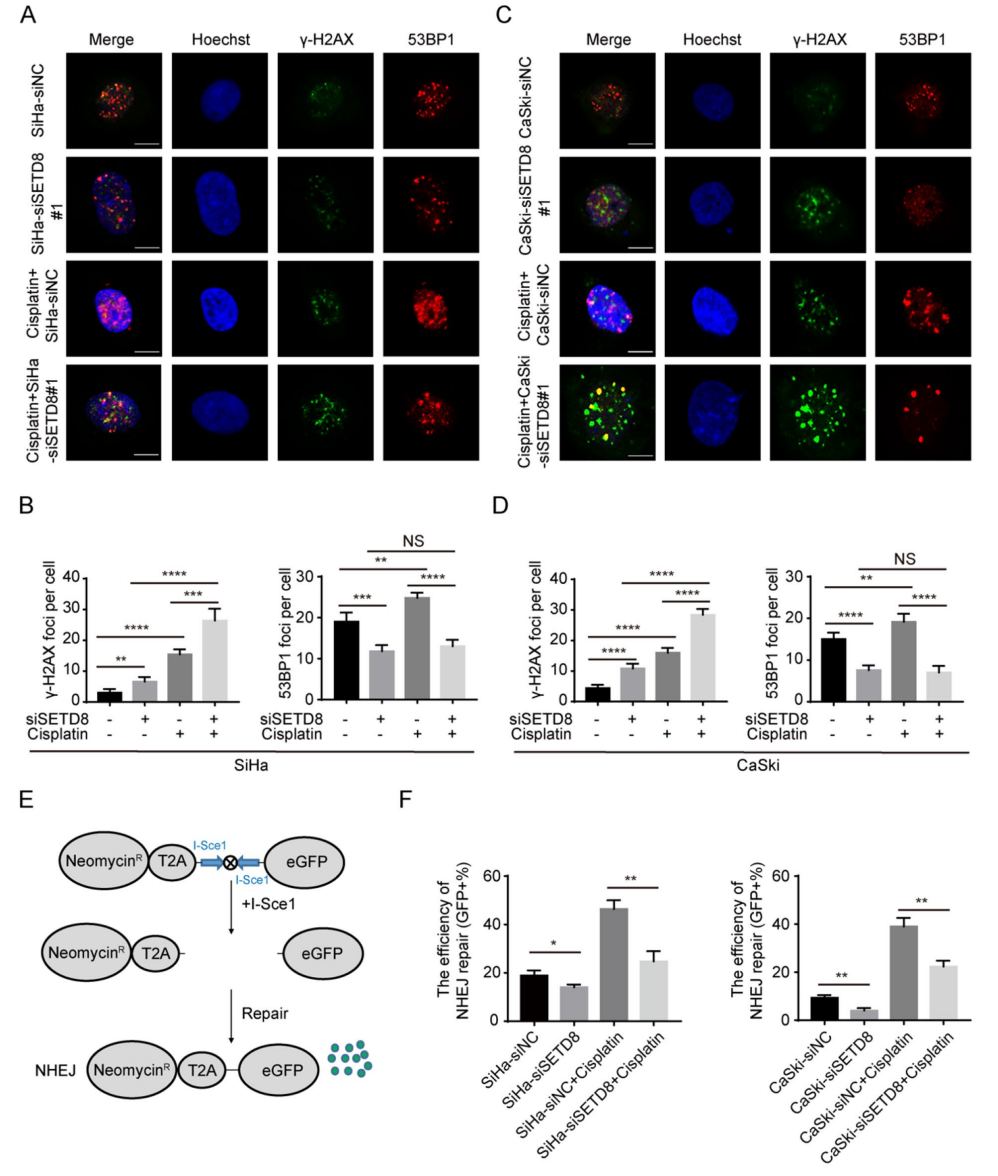
Reduction of 53BP1 binding to DNA breaks induced by *SETD8* downregulation leads to cisplatin sensitivity by inhibiting NHEJ. (**A-B**) Accumulation of *γ-*H2AX and 53BP1 following *SETD8* silencing in cisplatin-treated SiHa cells for 24 h and quantification of *γ-*H2AX and 53BP1 foci. (**C-D**) Accumulation of *γ-*H2AX and 53BP1 following *SETD8* silencing in cisplatin-treated CaSki cells for 24 h and quantification of *γ-*H2AX and 53BP1 foci. (**E**) Schematic diagram for DSB Repair Reporter. (**F**) NHEJ efficiency detected by DSB Repair Reporter. Cells with or without *SETD8* siRNA were treated with cisplatin for 24 h. *p* values were determined by two-tailed Student’s *t* test (ns: not significant; *: 0.01 ≤ *p* < 0.05; **: 0.001 ≤ *p* < 0.01; ***: 0.0001 ≤ *p* < 0.001; ****: *p* < 0.0001)

During further investigations, immunofluorescence was performed using 53BP1 and the DNA fragmentation marker *γ-*H2AX. As shown in Figs. 4A, 53BP1 foci were significantly reduced in *SETD8* knock-down SiHa cells (without cisplatin treatment: 19.00 ± 1.00 to 11.80 ± 0.66; *p* = 0.0003; with cisplatin treatment: 24.80 ± 0.58 to 13.00 ± 0.71; *p* < 0.0001) and were accompanied by an increase in *γ-*H2AX foci (without cisplatin treatment: 3.00 ± 0.55 to 6.60 ± 0.68; *p* = 0.0033; with cisplatin treatment: 15.40 ± 0.75 to 26.40 ± 1.72; *p* = 0.0004) (Fig. 4B). Similarly, Fig. 4C shows that knockdown of *SETD8* in CaSki cells also decreases 53BP1 foci (without cisplatin treatment: 15.00 ± 0.71 to 7.60 ± 0.51; *p* < 0.0001; with cisplatin treatment: 19.20 ± 0.86 to 7.00 ± 0.71; *p* < 0.0001) and increases *γ-*H2AX foci (without cisplatin treatment: 4.4 ± 0.51 to 10.80 ± 0.73; *p* < 0.0001; with cisplatin treatment: 16.00 ± 0.71 to 28.40 ± 0.87; *p* < 0.0001) (Fig. 4D).

On treatment of cells with cisplatin, more double strand DNA breaks (DSBs) are produced. The DSBs result in upregulation of NHEJ machinery and lead to efficient repair of the induced break [31, 32]. Therefore, we next set out to explore whether binding of 53BP1 to DSBs affected the NHEJ DNA repair pathway in cervical cancer cells. A DNA repair fluorescence reporter plasmid was used to assay cells repaired by NHEJ based on GFP expression (Fig. 4E). Flow cytometry was performed to detect GFP-positive cells. The proportion of GFP-positive cells allows estimation of the proportion of cells undergoing NHEJ repair. After knockdown of *SETD8*, the proportion of GFP-positive SiHa cells was significantly decreased (without cisplatin treatment: 18.87% ± 1.26 to 14.04% ± 0.66%, *p* = 0.0273; with cisplatin treatment: 46.36% ± 2.12 to 24.71% ± 2.46%, *p* = 0.0026) as was the proportion of GFP-positive CaSki cells (without cisplatin treatment: 9.33% ± 0.67 to 4.00% ± 0.58%, *p* = 0.0040; with cisplatin treatment: 39.00% ± 2.08 to 22.33% ± 1.45%, *p* = 0.0041) (Fig. 4F). Taken altogether, our data suggest that down-regulation of *SETD8* inhibits NHEJ by reducing the 53BP1 foci and leads to impaired repair of DNA strand breaks and improved sensitivity to cisplatin.

### *SETD8* expression negatively correlates with cisplatin sensitivity of primary tumor tissues and with clinical outcome in cervical cancer

**Fig. 5:**
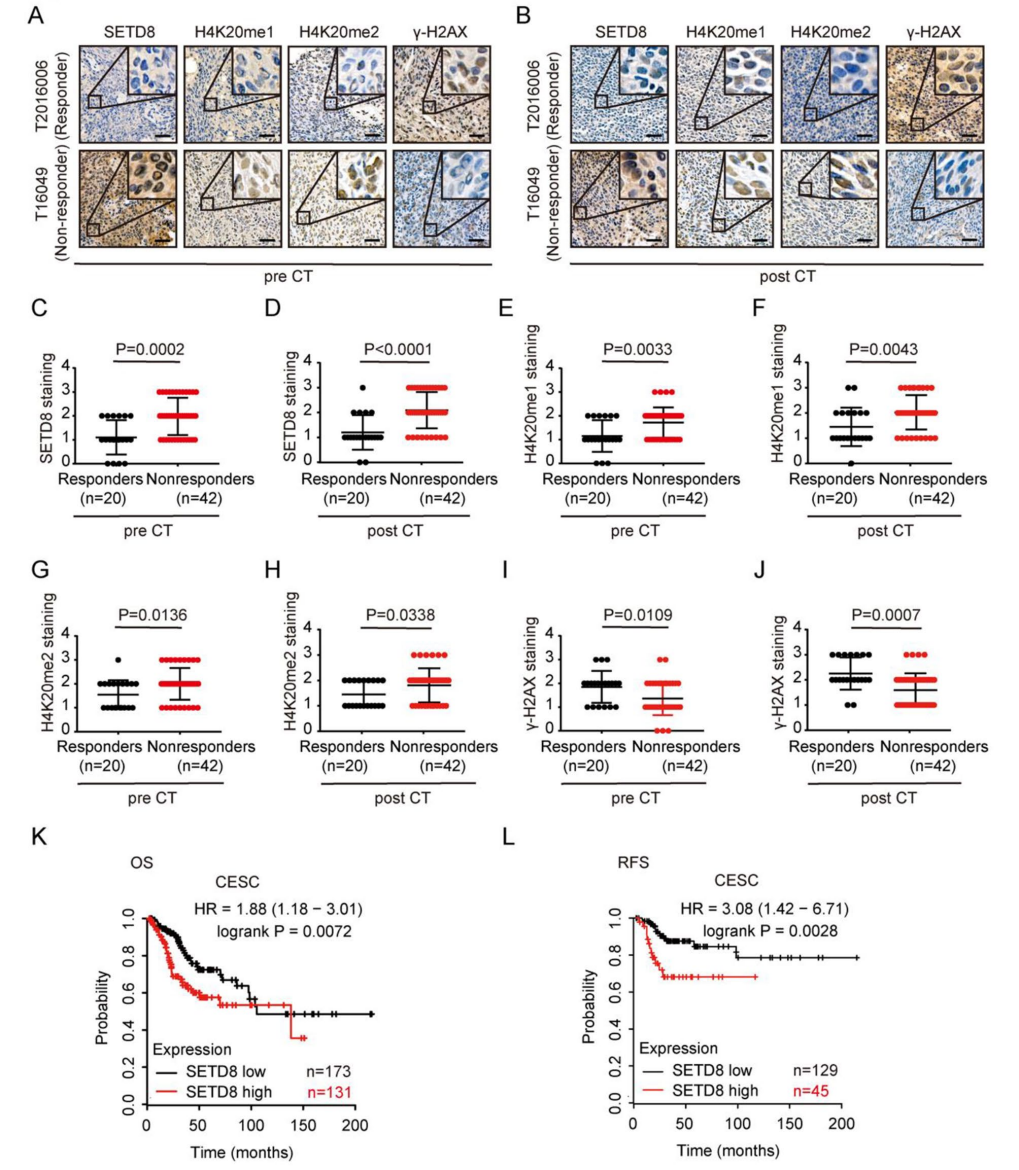
Inhibition of *SETD8* expression and H4K20 methylation are associated with cisplatin sensitivity and good clinical outcome in human cervical cancer. (**A**) *SETD8* and H4K20 methylation and *γ-H2AX* expression levels in pre-CT specimens of representative responder and non-responder detected by IHC staining. Images were taken at a magnification of 100× and 400× (insets). Scale bars: 10 μm. (**B**) *SETD8* and H4K20 methylation and *γ-H2AX* expression levels in post-CT specimens of representative responder and non-responder. (**C-J**) *SETD8* and H4K20 methylation level and *γ-H2AX* expression and drug response in specimens before (pre-CT) and after (post-CT) platinum-based chemotherapy. (**K-L**) Kaplan-Meier plotter analysis of cervical cancer patient groups. Patients were dichotomized by *SETD8* expression level at auto select best cutoff. Error bars represent ± SD. *p* values were determined by Mann-Whitney U test for (C-J), (ns: not significant; *: 0.01 ≤ *p* < 0.05; **: 0.001 ≤ *p* < 0.01; ***: 0.0001 ≤ *p* < 0.001; ****: *p* < 0.0001), log-rank test for (K-L).

We conducted IHC staining in 62 cervical cancer patients in receipt of platinum-based chemotherapy to investigate the association between *SETD8* expression and cisplatin-sensitivity (Fig. 5A-B). We found that the expression levels of *SETD8* were lower (pre-CT: *p* = 0.0002; post-CT: *p* < 0.0001) in the chemotherapy-responders compared with non-responders before and after chemotherapy (Fig. 5C, D). Furthermore, in pre-chemotherapy (pre-CT) and post-chemotherapy (post-CT) specimens, we observed that expression levels of H4K20me1 (pre-CT: *p* = 0.0033; post-CT: *p* = 0.0043) (Fig. 5E, F) and H4K20me2(pre-CT: *p* = 0.0136; post-CT: *p* = 0.0338) (Fig. 5G, H) were decreased and that of *γ-*H2AX (pre-CT: *p* = 0.0109; post-CT: *p* = 0.0007) (Fig. 5I, J) was increased in the chemotherapy-responders compared with non-responders. *SETD8* IHC staining was decreased in *SETD8* mutant samples compared with non-mutant samples, although this difference did not reach statistical significance (*p* = 0.1365) (supplementary Fig. S3A-B).

To demonstrate the clinical relevance of our findings, we evaluated the correlation of *SETD8*, H4K20me1, H4K20me2 and *γ-H2AX* levels in 62 cervical cancer specimens. We found that the expression level of *SETD8* was positively correlated with the H4K20me1 (*p* < 0.0001) and H4K20me2 (*p* < 0.0001) levels in pre-CT and post-CT specimens (supplementary Fig. S3C-D, E-F). Conversely, the expression level of *SETD8* was negatively correlated with the *γ-H2AX* level (pre-CT: *p* = 0.0005; post-CT: *p* < 0.0001) in pre-CT and post-CT specimens (supplementary Fig. S3G-H). These results are consistent with those obtained from experiments using cultured cells.

To explore the clinical relevance of our findings, we investigated whether the expression of *SETD8* can affect the prognosis of cervical cancer patients. Through the Kaplan-Meier Plotter database (https://kmplot.com/) [33], we found that the overall survival rates (OS) (HR = 1.88 [1.18–3.01], log-rank *p* = 0.0072) and recurrence-free survival rates (RFS) (HR = 3.08 [1.42–6.71], log-rank *p* = 0.0028) of the high *SETD8* expression patients were lower than those for patients with low *SETD8* expression (Fig. 5K-L). The data suggest that *SETD8* expression correlates negatively with cisplatin sensitivity and low *SETD8* expression could improve the patients’ prognoses.

### *SETD8* inhibitor UNC0379 improved cisplatin sensitivity in cervical cancer in vivo and in vitro

**Fig. 6:**
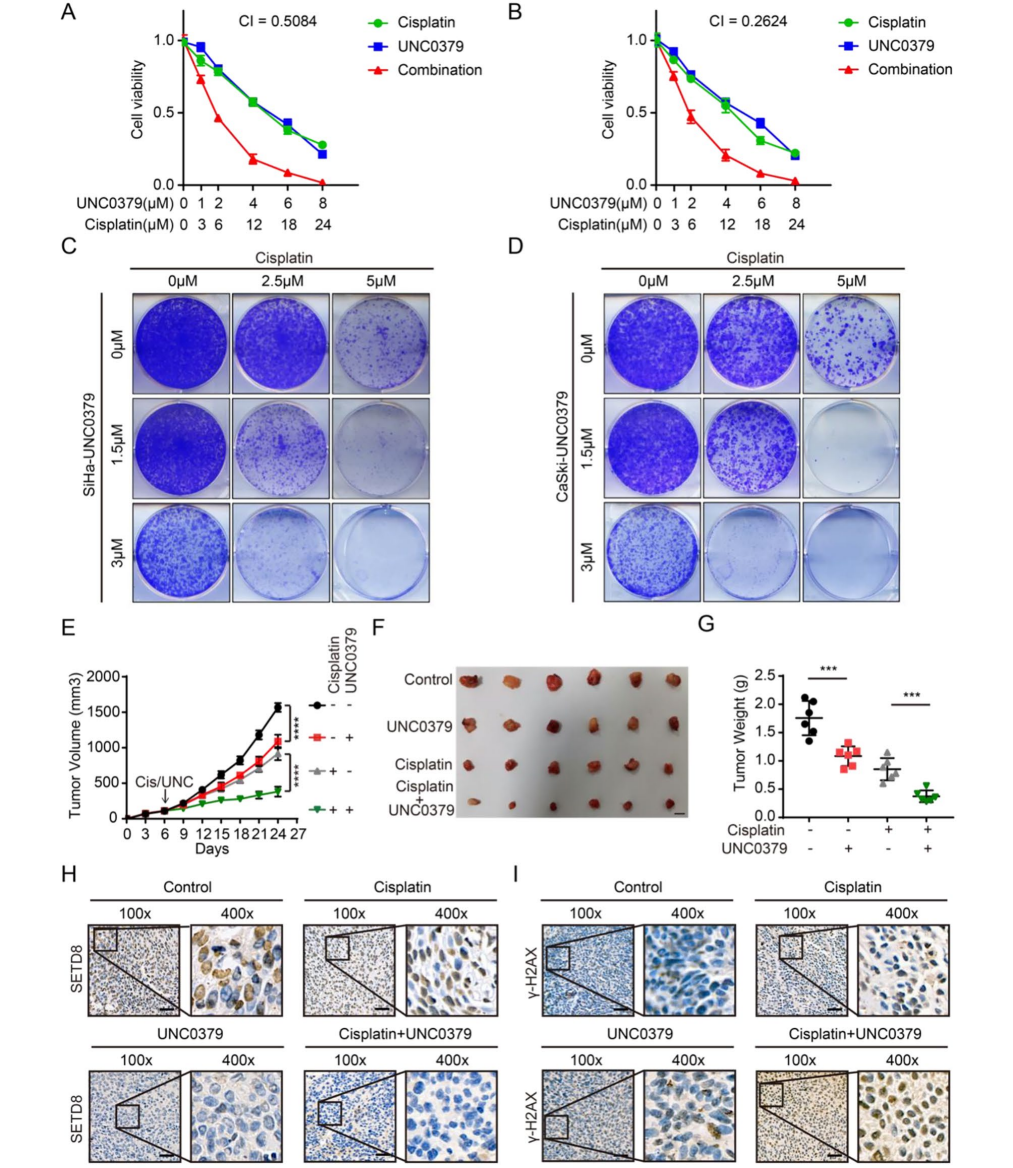
UNC0379 sensitizes cancer cells to cisplatin treatmentin vitro and in vivo. (**A-B**) dose-response curves for UNC0379 or cisplatin alone or combined in SiHa and CaSki cells treated with varying concentrations of UNC0379 (0–8 µM) and cisplatin (0–24 µM) with constant ratio of 1:3 when combined for 48 h. (**C-D**) Colony formation assays were performed using SiHa and CaSki cells with UNC0379 and cisplatin treatment. (**E**) Tumor growth of UNC0379- and cisplatin-treated mice carrying SiHa cell xenografts. Error bars represent SEM. (**F**) Size comparisons for tumors from each treatment group. Scale bar represents 10 mm. (**G**) Tumor weight for each group, *p* values were determined by unpaired Student’s *t* test; error bars represent SD. (**H**) *SETD8* IHC staining of subcutaneous tumors of each group at the experimental endpoint. Images were taken at a magnification of 100× and 400×. Scale bars represent 10 μm. (**I**) *γ-H2AX* IHC staining at the experimental endpoint. Images were taken at a magnification of 100× and 400×. Scale bars represent 10 μm

Inhibition of *SETD8* improves the sensitivity of cancer cells to cisplatin indicating that *SETD8* may be an anticancer target. UNC0379 has been identified as an inhibitor of *SETD8* [34]. Dose-response curves of UNC0379 (0–8 µM), cisplatin (0–24 µM) and their combinations (ratio, 1:3) were constructed in SiHa and CaSki cells over a 48 h period. A combination index (CI) was calculated using CompuSyn software with the Chou-Talalay equation [35] which allows the definition of an additive effect (CI = 1), synergism (CI < 1) or antagonism (CI > 1) in drug combinations. We found that UNC0379 demonstrated synergy with cisplatin in SiHa cells (CI = 0.5084) (Fig. 6A) and in CaSki cells (CI = 0.2624) (Fig. 6B). In addition, UNC0379 greatly increased cisplatin-induced apoptosis in SiHa cells (7.11% ± 2.67 to 83.11% ± 7.11%; *p* < 0.0001) and in CaSki cells (31.25% ± 2.92 to 59.58% ± 4.58%; *p* = 0.0008) (supplementary Fig. S4A-B). In the presence or absence of cisplatin, UNC0379 inhibited NHEJ repair as detected by DNA fluorescence reporter plasmid in both SiHa cells (without cisplatin treatment: 10.20% ± 1.64 to 5.70% ± 1.91%, *p* = 0.0366; with cisplatin treatment: 56.36% ± 6.33 to 29.71% ± 6.22%, *p* = 0.0065) and in CaSki cells (without cisplatin treatment: 12.67% ± 2.52 to 7.33% ± 1.16%, *p* = 0.0289; with cisplatin treatment: 65.67% ± 4.04 to 31.67% ± 3.51%, *p* = 0.0004) (supplementary Fig. S4C-D). The combination of UNC0379 (0 µM, 1.5 µM, 3 µM) and cisplatin (0 µM, 2.5 µM, 5 µM) produced enhanced reduction of colony formation of SiHa and CaSki cells compared with cisplatin alone. Moreover, UNC0379 alone inhibited colony formation of SiHa and CaSki cells (Fig. 6C-D).

We verified the sensitizing effect of UNC0379 on cisplatin using a mouse model of a subcutaneous tumor formed from SiHa cells. Our results demonstrate that the combination of UNC0379 and cisplatin significantly reduced tumor size (*p* < 0.0001) compared with cisplatin monotherapy and showed an improvement in therapeutic effect (Fig. 6E-F). The combination of UNC0379 and cisplatin led to a significant reduction in tumor weight (*p* = 0.0004) compared to cisplatin monotherapy (Fig. 6G). Via IHC staining of the subcutaneous tumor [36], we were able to demonstrate that UNC0379 also reduced SETD8 protein levels (Fig. 6H; supplementary Fig. S4E-F). Moreover, the combination of UNC0379 and cisplatin substantially increased levels of *γ-H2AX* protein (Fig. 6I, supplementary Fig. S4G-H) in subcutaneous tumor tissues, indicating an increase in DNA breaks. Together, data from in vitro and in vivo experiments suggested that UNC0379 enhanced the therapeutic effects of cisplatin.

**Fig. 7:**
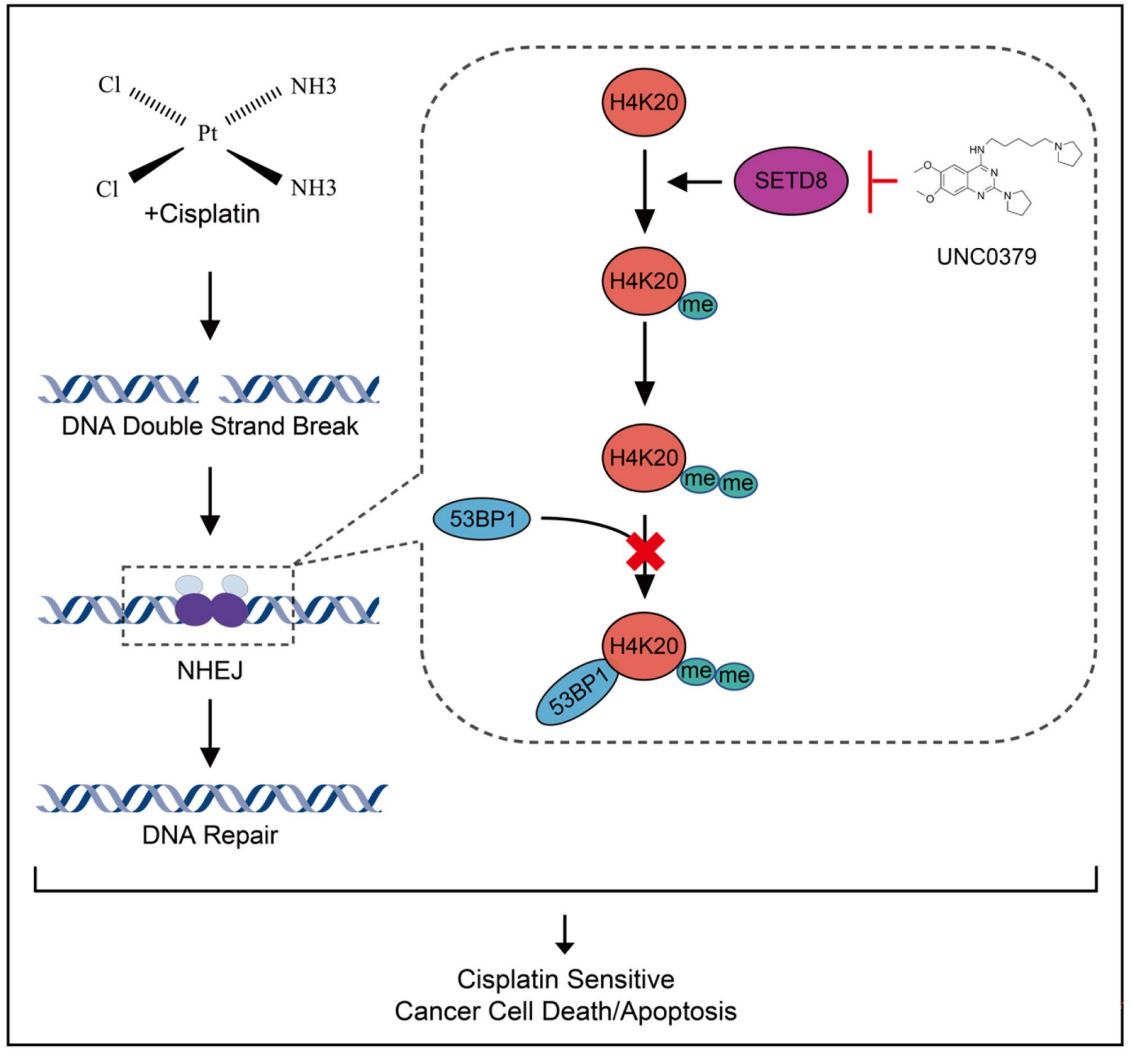
Schematic diagram of SETD8 inhibition sensitizes cervical cancer cells to cisplatin treatment. Downregulation of SETD8 significantly reduces the mono-methylation level of H4K20 (H4K20me1) and double-methylation level of H4K20 (H4K20me2) in cervical cancer and further reduces the recruitment of the DNA damage repair factor 53BP1, inhibits the non-homologous end joining repair pathway (NHEJ), resulting in increased cisplatin-induced DNA damage

## Discussion

WES of 156 cervical cancers has allowed us to identify a frequently mutated gene *SETD8*, which enhances the chemotherapeutic efficacy. Inhibition of *SETD8* decreased the methylation level of H4K20, leading to reduced binding of 53BP1 to the DSBs caused by cisplatin treatment. Lack of recruitment of 53BP1 to DNA strand breaks inhibited NHEJ, resulting in increased frequency of DNA breaks and apoptosis in the cisplatin-treated cell (Fig. 7). In agreement with this proposed mechanism, we found that downregulation of *SETD8* in clinical samples correlated positively with sensitivity to platinum-based therapy and improved prognosis of cervical cancer patients. Our data provides new insights into the prediction and the prevention of cisplatin resistance.

Increased DNA repair accounts for a significant proportion of cisplatin resistance [37]. Therefore, DNA repair pathways are promising targets for cancer treatment. Such approach may be used to sensitize cancer cells in chemo/radiation therapy since DSBs are the most lethal form of DNA lesions [38]. Approximately five DNA repair pathways exist in mammalian cells: mismatch repair (MMR), nucleotide excision repair (NER), base excision repair (BER), homologous recombination (HR) and non-homologous end joining (NHEJ) [39, 40]. Many pathways are already targeted for the treatment of cancer. For example, *PARP* inhibitors have been approved to treat *BRCA1/BRCA2* mutated tumors by targeting homologous recombination deficiency [41].

In addition, drugs with epigenetic effects have been found to modulate DSB repair [42, 43] and may serve as anti-cancer drugs. The current study indicated that *SETD8*, which encodes a mono-methyltransferase acting on H4K20, could be used as a new therapeutic target for drug resistance in cervical cancer. We identified UNC0379 as a *SETD8* inhibitor with promising therapeutic potential. UNC0379 increased cisplatin sensitivity both in cultured cells and in a mouse model of cervical cancer. Sensitization has been reported as one of the important mechanisms of synergistic effect [44–46]. In our study, we found that UNC0379 enhances the sensitization of cisplatin (supplementary Fig. 6A-B) by inhibiting the NHEJ pathway (supplementary Fig. S4C-D and S5A-B). Moreover, we found that the IC50 for cisplatin on breast and ovarian cancer cell-lines in the GDSC database depends on the expression level of *SETD8* (https://www.cancerrxgene.org/, data access date: July 2019) [47] (supplementary Fig. S4I-J). In addition, levels of *SETD8* expression related to the OS (HR = 1.41 [1.14–1.74], logrank *p* = 0.0017) and RFS (HR = 1.26 [1.12–1.42], logrank *p* = 8.8 × 10^− 5^) of breast cancer patients in the Kaplan-Meier Plotter database [33] (supplementary Fig. S4K-L). We believe *SETD8*-targeted therapy may have values for patients with resistance to platinum-based chemotherapy in various cancers.

## Conclusions

*SETD8* could constitute a predictive marker for cancer outcome and is a promising therapeutic target to treat resistance to platinum-based therapy. A small molecule inhibitor of *SETD8*, UNC0379, could act as a cisplatin-sensitizer in cervical cancer. Our results indicate the necessity of additional pre-clinical and clinical studies which may lead to improved treatment for cisplatin resistance in cancer patients.

## Methods

### Whole exome sequencing

Samples of patient tumor tissue were collected before platinum-based NACT. Biopsies were collected and fixed with formalin and paraffin-embedded (FFPE), which were used by expert pathologists for histological identification of cervical squamous cell carcinoma. Paired samples of normal tissue and tumor tissue DNA were sequenced by WES on an Illumina HiseqX platform with paired-end reads of 150 bp. Sequence reads were analyzed according to GATK best practice [48]. Paired-end reads were mapped to the reference genome (UCSC hg19) with BWA-MEM (v0.7.8) [49]. Picard tools were employed to mark PCR duplicate reads and the Indel Realigner algorithm (GATK v3.8.0) was used to improve alignment accuracy. The MuTect2 (GATK v3.8.0) [50] was used to detect somatic mutations in paired samples of tumor DNA versus control. Somatic mutations with an allele frequency of lower than 5% were filtered out [51, 52]. High confidence variants were annotated with ANNOVAR (v2015Mar22) [53]. To control for possible germline contamination, somatic SNVs and indels with a population frequency of greater than 1% in 1000G/EXAC/ESP6500 [54] were filtered. Capture libraries were sequenced on the Illumina HiseqX platform with a mean coverage of 200×.

### NACT clinical response

The clinical response to NACT was determined by measuring dynamic changes in tumor volume during each cycle of treatment. Tumor remission was defined by the clinical response criteria of the World Health Organization (WHO) [55]. A decrease in tumor size of more than 50% was defined as a responder while a decrease in tumor size of less than 50% or an increase in tumor size or emergence of new lesions was defined as a non-responder.

### Cell culture and reagents

SiHa, CaSki cell lines were obtained from the American Type Culture Collection (ATCC). SiHa, CaSki cell lines were cultured in Dulbecco’s modified Eagle medium (DMEM, Gibco) with 10% fetal bovine serum (Every Green). The Mycoplasma Stain Assay Kit (Beyotime) was used for *Mycoplasma* test of cell lines and authenticate of cell lines were verified by short tandem repeat (STR) profiling. UNC0379, a small molecule inhibitor of *SETD8*, was obtained from Selleck (S7570).

### siRNA, cell viability, colony formation and apoptosis assays

Specific siRNA for *SETD8* was obtained from Ribobio (target sequence: CCTAGGAAGACTGATCAATC). SiHa, CaSki, MCF-7 and SKOV3 cells were transfected with siRNA using lipofectamine 3000 (Invitrogen, L3000015) in 6-well plates for 24 h before seeding into 96-well plates (4 replicates per condition). 48 h after transfection, the cells were treated with cisplatin. Cell viability was determined after 2 days of cisplatin treatment using a cell counting kit-8 (Dojindo, CK04), according to the manufacturer’s instructions. For the colony formation assay, 200–500 cells/well were seeded in 12-well plates and treated with PBS and cisplatin for 48 h. The colonies were stained with 0.5% crystal violet and counted by ImageJ software after 12 days. Apoptotic cells were assessed using a FITC Annexin V apoptosis detection kit (Vazyme, #A211-02).

### Immunoblotting analysis

Cells were lysed in SDS lysis buffer. After heating for 10 min at 100℃, 50 µg or the indicated amount of protein extract was loaded onto SDS-PAGE, followed by transfer to PVDF membranes. Membranes were blocked with 5% milk-TBST and incubated with primary antibodies against the following proteins: *SETD8* (Proteintech, 14063-1-AP, 1:500), H4K20me1 (Abclonal, A2370, 1:1000), H4K20me2 (Abclonal, A2371, 1:1000), GAPDH (Abclonal, AC001, 1:20000), 53BP1 (Abclonal, A5757, 1:500), and *γ-*H2AX (Abclonal, AP0245, 1:1000). Membranes were incubated with the following secondary antibodies: HRP goat anti-mouse (antGene, ANT019, 1:6000) or HRP goat anti-rabbit (antGene, ANT020, 1:6000).

### Immunofluorescence staining

Transfected or treated cells cultured on glass coverslips were washed with PBS and fixed with 4% paraformaldehyde for 15 min at room temperature. Cells were incubated with agitation in PBS containing 5% BSA for 1 h, followed by incubation with a primary mouse anti-*γ-*H2AX antibody (Abclonal, AP0245, 1:100) and a primary rabbit anti-53BP1 antibody (Abclonal, A5757, 1:100) overnight at 4℃ and with secondary antibodies conjugated with Alexa-488 (antGene, ANT023, 1:200) in PBS for 1 h at room temperature. Excess antibody was washed away by PBST followed by incubation with secondary antibodies conjugated with Alexa-594 (antGene, ANT030, 1:200) in PBS for 1 h at room temperature. Slides were counterstained with 4 µg/ml Hoechst33258 (servicebio, G1011) in glycerol. Confocal laser-scanning immunofluorescence microscopy was performed by Generulor Company Bio-x Lab, wuhan, hubei, China. Images were assembled using Adobe Photoshop CC. Quantitative analysis was performed by inspecting the cells from three separate experiments. Values are expressed as mean ± sd.

### Double strand break repair reporter (DRR)

The Double Strand Break (DSB) repair reporter plasmid was obtained from Addgene (#98,895). The integrated DRR consists of a promoter and a resistance cassette fused to a T2A peptide and two inverted ISce1 sites followed by GFP. Intact or partially cut DRR lacks GFP expression due to the presence of a stop codon. Cells repaired by NHEJ express GFP.

### Immunohistochemistry

FFPE tumor tissue sections underwent antigen retrieval, endogenous peroxidase blocking and incubation with a primary antibody overnight at 4℃. Immunohistochemistry **(**IHC) secondary staining involved an HRP-conjugated goat anti-rabbit or goat anti-mouse secondary antibody (1:250) and the signals were detected using DAB reagent. Quantitative analysis was performed with ImageJ software. Positive staining of the tumor cells was identified using IHC signal intensity scored from 0 to 3.

### Drug assays

*SETD8* inhibitor, UNC0379, (Selleck, S7570) was suspended at a 50 mM stock concentration in DMSO. Cisplatin (Solarbio, D8810) was suspended at a 10 mM stock concentration in double distilled water (ddH2O). Cells were cultured as described above, seeded at 4,000 cells per well of a 96-well plate and incubated for 24 h to ensure adherence. UNC0379 was initially diluted to a 1 mM concentration in opti-MEM. Cisplatin was initially diluted to a 100 µM concentration in opti-MEM. The 100 µM solution was used to prepare solutions ranging from 0.01 to 100 µM concentration. For the cisplatin + UNC0379 experiment, the cisplatin IC50 concentration in a cell line was calculated and UNC0379 was initially added for 24 h before being re-added in combination with the cisplatin dilution series prepared as described above for 48 h. Data analysis of the drug inhibitor assays was performed using GraphPad Prism 7 (San Diego, CA). Data were fitted to obtain the concentration-response curves using a four-parameter logistic equation (for IC50 values). Statistical differences were analyzed using Student’s *t*-test and *p* < 0.05 was considered significant.

### Quantitative RT-PCR

Cells were transfected with siRNA and then treated with ddH2O/cisplatin for 48 h before extraction of RNA using a total RNA kit I (Omega Bio-tek, R6834-01). Sample mRNA was quantitatively analyzed by qRT-PCR using an iTaqTM Universal SYBR Green Supermix (Bio-Rad, #1725125) and a 7500 real-time PCR instrument (Applied Biosystems). The primer sequences were as follows: *SETD8*, forward primer: 5’-ACAAATGCTCTGGAATGCGTT-3’; reverse primer: 5’-CCGGCTAATGGTTTCCCCTG-3’; and *GAPDH*, forward primer: 5’-AATGGACAACTGGTCGTGGAC-3’; reverse primer: 5’-CCCTCCAGGGGATCTGTTTG-3’. Primer synthesis was performed by TSINGKE Biotech.

### Xenograft tumor assays

Five million SiHa cells were suspended in 20% Matrigel (BD Biosciences) in PBS. The mixture was subcutaneously injected into 6–week-old BALB/c null mice. Treatments were administered as follows: cisplatin (5 mg/kg twice a week by intraperitoneal injection) and UNC0379 (5 mg/kg 3 times a week by subcutaneous injection) from day 6 after xenograft initiation for a total of 18 days. Tumor growth was recorded by blind measurement of two perpendicular diameters of the tumor and tumor volume calculated using the equation: 4π/3 × (width/2)^2^ × (length/2). Tumors were harvested at the experimental endpoint. Animals were randomly selected for all animal studies. Concealed allocation and blinding of the outcome assessment were used.

### Statistics

All experiments were repeated at least three times and the data are presented as the mean ± standard deviation (SD) or the mean ± standard error of the mean (SEM). The statistical analyses were performed using GraphPad Prism 7. Differences between two groups were analyzed by Student’s *t* test (two-sided) and Fisher’s exact test with significance set at *p* < 0.05. Specific details of statistical methods are given in the corresponding figure legends.

## Electronic supplementary material

Below is the link to the electronic supplementary material.

**Additional File 1: Figure S1.** TMB for *SETD8* WT and *SETD8* Mutant specimens in the WES cohort.

**Additional File 2: Figure S2.** 53BP1 recruitment decreased after *SETD8* knockdown in SiHa and CaSki with cisplatin treatment.

**Additional File 3: Figure S3.***SETD8* mutation can affect the methyltransferase activity on H4K20 and SETD8 correlated with H4K20me1/2 and γ-H2AX levels before and after cisplatin chemotherapy.

**Additional File 4: Figure S4.** UNC0379 increased apoptosis inducted by cisplatin and impaired NHEJ repair efficiency in vivo and in vitro.

**Additional File 5: Figure S5.** UNC0379 sensitizes cervical cancer cells to cisplatin treatment in vitro.

**Additional File 6: Table S1.** Patient characteristics of NACT Cohort.

**Additional File 7: Table S2.**

**Additional File 8: Table S3.** Analysis of *SETD8* mutations and predicting the functional effects by silico analysis.

**Additional File 9: Table S4.**

**Additional File 10:** Original Western Blots.

## Data Availability

All data needed to evaluate the conclusions in the paper are present in the paper and the Supplementary Materials.

## Abbreviations

WES: whole exome sequencing
NHEJ: non-homologous end joining
H4K20me1: mono-methylation of H4K20
p53K382me1: mono-methylation of p53
*PCNAK248me1*: mono-methylate *PCNA* on lysine 248
TMB: tumor mutational burden
NACT: neoadjuvant chemotherapy
DSB: double strand DNA breaks
