# Supplementary material for: SETD8, a frequently mutated gene in cervical cancer, enhances cisplatin sensitivity by impairing DNA repair
Source: Cell Biosci. 2023 Jun 12;13:107. doi: 10.1186/s13578-023-01054-y (PMC10262521; doi:10.1186/s13578-023-01054-y)
Supplement: Supplementary file 1 — Additional File 1: Figure S1. TMB for SETD8 WT and SETD8 Mutant specimens in the WES cohort. [file 13578_2023_1054_MOESM1_ESM.pdf]

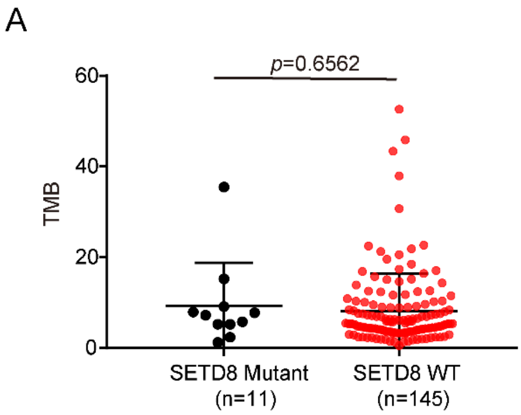

Figure S1. TMB for *SETD8* WT and *SETD8* Mutant specimens in the WES cohort.

(A) Statistics of TMB for *SETD8* WT and *SETD8* Mutant specimens. Error bars represent ± SD. *p* values were determined by unpaired Student's *t* test.
