# Supplementary material for: SETD8, a frequently mutated gene in cervical cancer, enhances cisplatin sensitivity by impairing DNA repair
Source: Cell Biosci. 2023 Jun 12;13:107. doi: 10.1186/s13578-023-01054-y (PMC10262521; doi:10.1186/s13578-023-01054-y)
Supplement: Supplementary file 2 — Additional File 2: Figure S2. 53BP1 recruitment decreased after SETD8 knockdown in SiHa and CaSki with cisplatin treatment. [file 13578_2023_1054_MOESM2_ESM.pdf]

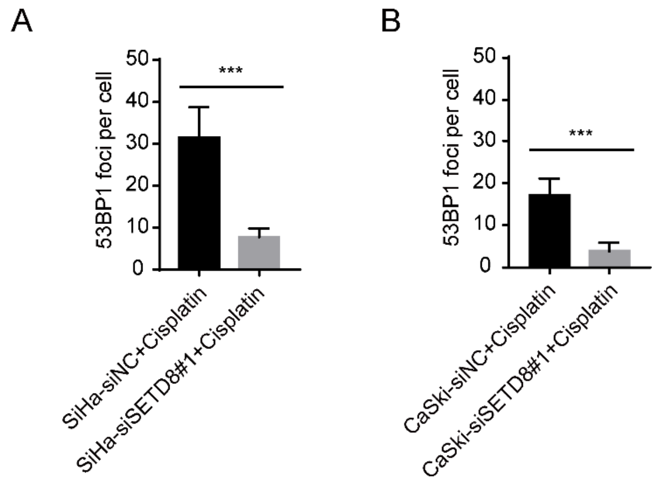

Figure S2. 53BP1 recruitment decreased after *SETD8* knockdown in SiHa and CaSki with cisplatin treatment.

(A). Quantification of 53BP1 foci after *SETD8* knockdown in cisplatin-treated SiHa; (B). Quantification of 53BP1 foci after *SETD8* knockdown in cisplatin-treated CaSki.
