# Supplementary material for: SETD8, a frequently mutated gene in cervical cancer, enhances cisplatin sensitivity by impairing DNA repair
Source: Cell Biosci. 2023 Jun 12;13:107. doi: 10.1186/s13578-023-01054-y (PMC10262521; doi:10.1186/s13578-023-01054-y)
Supplement: Supplementary file 3 — Additional File 3: Figure S3.SETD8 mutation can affect the methyltransferase activity on H4K20 and SETD8 correlated with H4K20me1/2 and γ-H2AX levels before and after cisplatin chemotherapy. [file 13578_2023_1054_MOESM3_ESM.pdf]

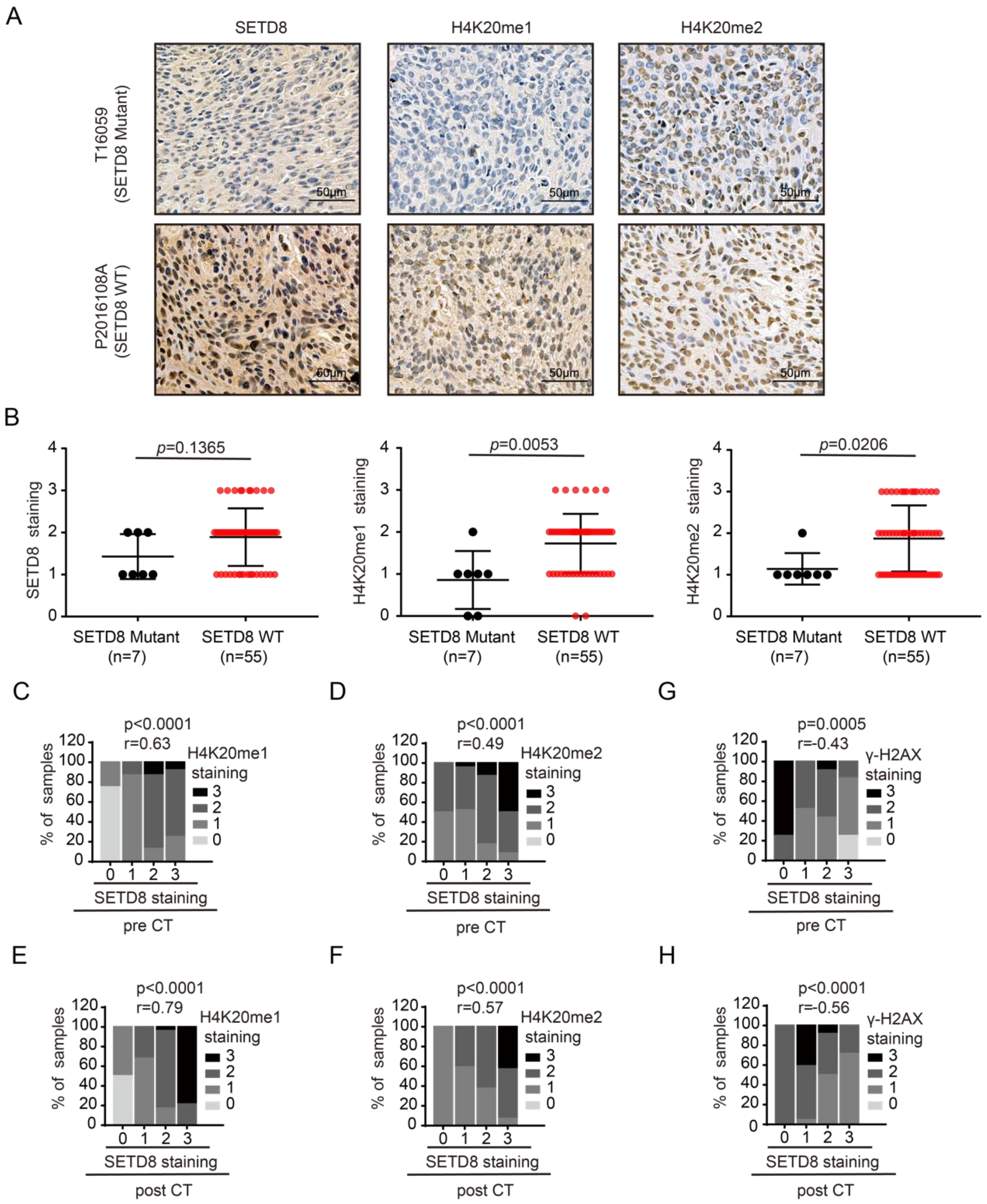

Figure S3. *SETD8* mutation can affect the methyltransferase activity on H4K20 and SETD8 correlated with H4K20me1/2 and γ-H2AX levels before and after cisplatin chemotherapy.

(A) *SETD8*, *H4K20me1* and *H4K20me2* expression levels in specimens of representative *SETD8* WT and *SETD8* Mutant detected by IHC. Images were taken at a magnification of 400×. Scale bars: 50μm; (B) Statistics of *SETD8*, *H4K20me1* and *H4K20me2* expression levels in *SETD8* WT and *SETD8* Mutant specimens. Error bars represent ± SD. *p* values were determined by Mann-Whitney U test; (C-D, G). SETD8 correlated with H4K20me1/2 and

γ-H2AX levels before cisplatin chemotherapy; (E-F, H). SETD8 correlated with H4K20me1/2 and γ-H2AX levels after cisplatin chemotherapy.
