# Supplementary material for: SETD8, a frequently mutated gene in cervical cancer, enhances cisplatin sensitivity by impairing DNA repair
Source: Cell Biosci. 2023 Jun 12;13:107. doi: 10.1186/s13578-023-01054-y (PMC10262521; doi:10.1186/s13578-023-01054-y)
Supplement: Supplementary file 4 — Additional File 4: Figure S4. UNC0379 increased apoptosis inducted by cisplatin and impaired NHEJ repair efficiency in vivo and in vitro. [file 13578_2023_1054_MOESM4_ESM.pdf]

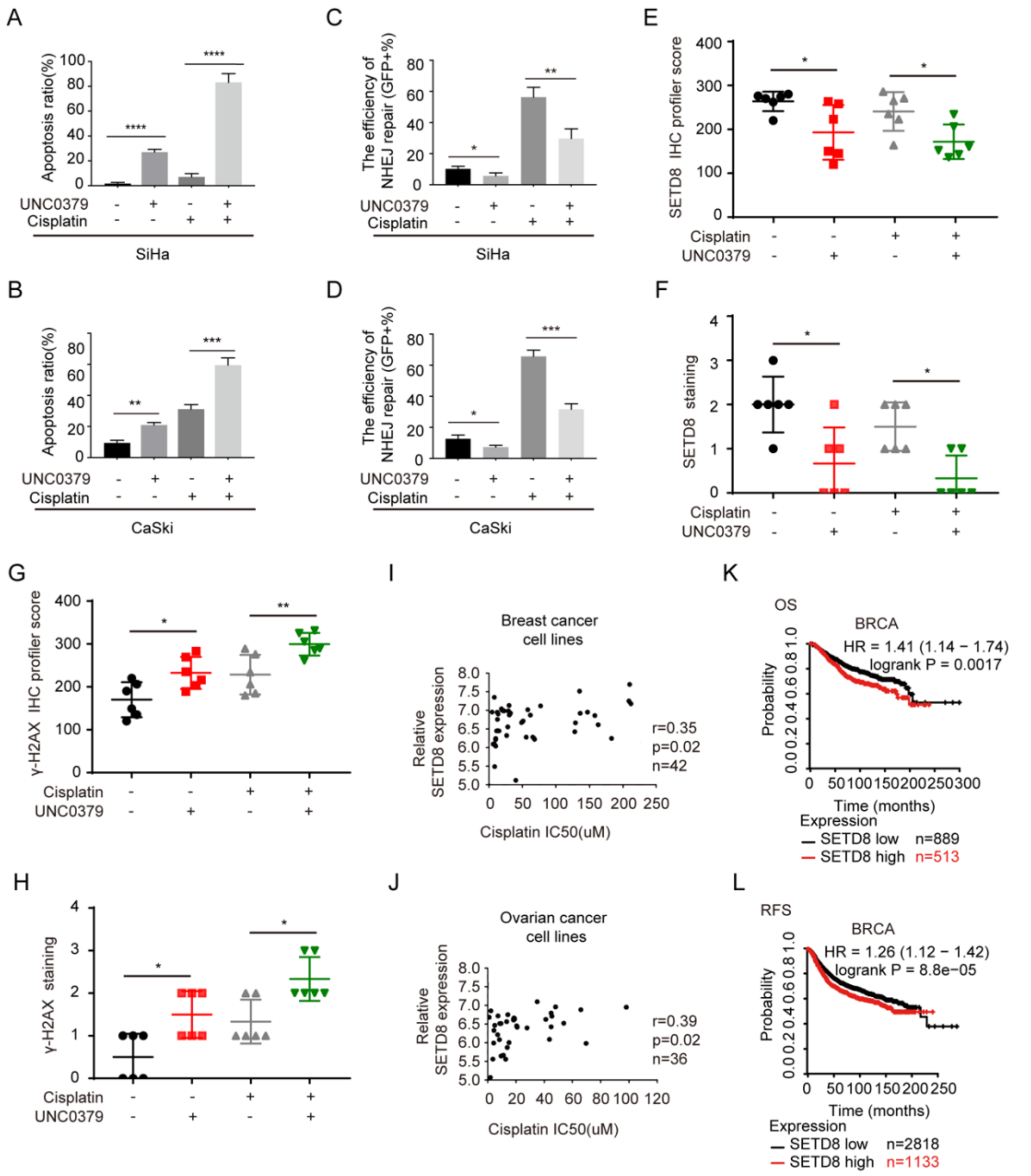

Figure S4. UNC0379 increased apoptosis inducted by cisplatin and impaired NHEJ repair efficiency *in vivo* and *in vitro*.

(A-D). Apoptosis assay using SiHa cells (A) and CaSki cells (B) with UNC0379 and cisplatin treatment. Cells were treated with cisplatin for 48 h and apoptotic cells were assayed by Annexin V staining. NHEJ efficiency detected by DSB Repair Reporter in SiHa (C) and CaSki (D) cells. Cells with or without UNC0379 were treated with cisplatin for 24 h. Error bars represent ± SD from three replicates. p values were determined by two-tailed Student's t test (ns: not significant; *: $0.01 \le p < 0.05$; **: $0.001 \le p < 0.01$; ***: $0.0001 \le p < 0.0001$). (E-H) Quantification of SETD8 (E) and γ-H2AX (G) IHC data using IHC profiler score in vehicle control,

UNC0379, cisplatin and the combination groups. p values were determined by two-tailed Student's t test. Quantification of SETD8 (F) and γ-H2AX (H) IHC data using staining intensity in vehicle control, UNC0379, cisplatin and the combination groups. p values were determined by Mann-Whitney U test. (I-L) The expression of SETD8 is positively correlated with the IC50 of cisplatin in breast cancer (I) and ovarian cancer (J) cell lines. KaplanMeier plotter analysis of OS (K) and RFS (L) in breast cancer patient groups. Patients were dichotomized by SETD8 expression level at auto select best cutoff.
