# Supplementary material for: SETD8, a frequently mutated gene in cervical cancer, enhances cisplatin sensitivity by impairing DNA repair
Source: Cell Biosci. 2023 Jun 12;13:107. doi: 10.1186/s13578-023-01054-y (PMC10262521; doi:10.1186/s13578-023-01054-y)
Supplement: Supplementary file 5 — Additional File 5: Figure S5. UNC0379 sensitizes cervical cancer cells to cisplatin treatment in vitro. [file 13578_2023_1054_MOESM5_ESM.pdf]

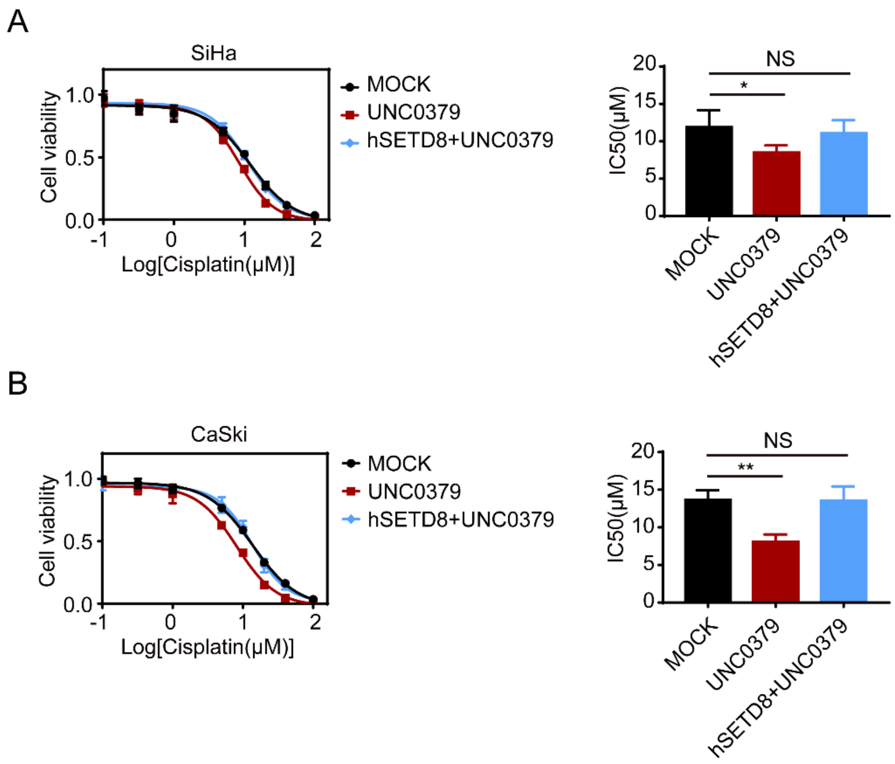

Figure S5. UNC0379 sensitizes cervical cancer cells to cisplatin treatment *in vitro*. (A) Cell viability and IC50 of cisplatin in SiHa cells with vehicle control, UNC0379, hSETD8+UNC0379. (B) Cell viability and IC50 of cisplatin in CaSki cells with vehicle control, UNC0379, hSETD8+UNC0379
