# Supplementary material for: SETD8, a frequently mutated gene in cervical cancer, enhances cisplatin sensitivity by impairing DNA repair
Source: Cell Biosci. 2023 Jun 12;13:107. doi: 10.1186/s13578-023-01054-y (PMC10262521; doi:10.1186/s13578-023-01054-y)
Supplement: Supplementary file 6 — Additional File 6: Table S1. Patient characteristics of NACT Cohort. [file 13578_2023_1054_MOESM6_ESM.docx]

|  |  |
| --- | --- |
|  | **NACT Cohort (n=156)** |
| **Age-yr** |  |
| ≥ 60 | 17 (10.90%) |
| < 60 | 139 (89.10%) |
| **Differentiation** |  |
| Well | 2 (1.28%) |
| Moderate | 67 (42.95%) |
| Poor | 62 (39.74%) |
| Unknown | 25 (16.03%) |
| **Stage** |  |
| IB1 | 7 (4.49%) |
| IB2 | 29 (18.59%) |
| IIA | 41 (26.28%) |
| IIB | 79 (50.64%) |
| **Mean Tumor diameter-cm** | 4.5 |
| **Lymph node metastasis** |  |
| Negative | 119 (76.28%) |
| Positive | 29 (18.59%) |
| Unknown | 8 (5.13%) |
| **Mean SCC-μg/L** | 8.3 |
| **Chemotherapy** |  |
| Response | 104 (66.67%) |
| Non-response | 52 (33.33%) |
| NOTE. Percentages may not total 100, because of rounding. | |
| **Table S1: Patient characteristics of NACT Cohort** | |
