# Supplementary material for: SETD8, a frequently mutated gene in cervical cancer, enhances cisplatin sensitivity by impairing DNA repair
Source: Cell Biosci. 2023 Jun 12;13:107. doi: 10.1186/s13578-023-01054-y (PMC10262521; doi:10.1186/s13578-023-01054-y)
Supplement: Supplementary file 8 — Additional File 8: Table S3. Analysis of SETD8 mutations and predicting the functional effects by silico analysis. [file 13578_2023_1054_MOESM8_ESM.docx]

| Missense mutations | Amino acid changes | Mutation patients | Polyphen2 | MutationTaster | CADD | Mutation Assessor |
| --- | --- | --- | --- | --- | --- | --- |
| 14_16del | - | 1 | - | poly-morphism | Benign | - |
| I152= | - | 1 | - | poly-morphism | Benign | - |
| A21V | conservative | 1 | Benign | poly-morphism | Benign | Neutral |
| P60L | conservative | 3 | Damaging | Disease causing | Deleterious | Low |
| K121R-G122R^†^ | Non-conservative | 1 | Damaging | Disease causing | Deleterious | Low |
| R238P | Non-conservative | 6 | Damaging | Disease causing | Deleterious | Low |
| R258W | Non-conservative | 1 | Damaging | Disease causing | Deleterious | High |
| ^†^The missense mutation K121R and G122R occur in the same patient. | | | | | | |

**Table S3. Analysis of *SETD8* mutations and predicting the functional effects by silico analysis**
