# Supplementary figures and images for: SETD8, a frequently mutated gene in cervical cancer, enhances cisplatin sensitivity by impairing DNA repair
Source: Cell Biosci. 2023 Jun 12;13:107. doi: 10.1186/s13578-023-01054-y (PMC10262521; doi:10.1186/s13578-023-01054-y)

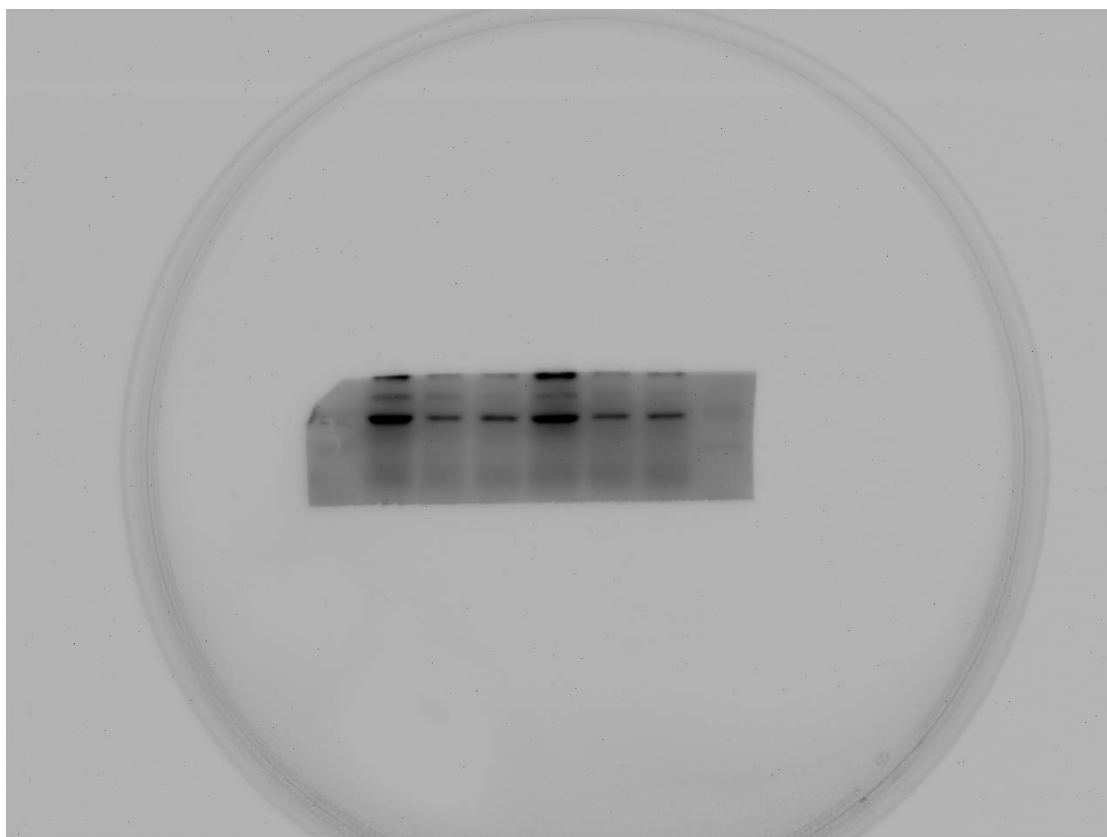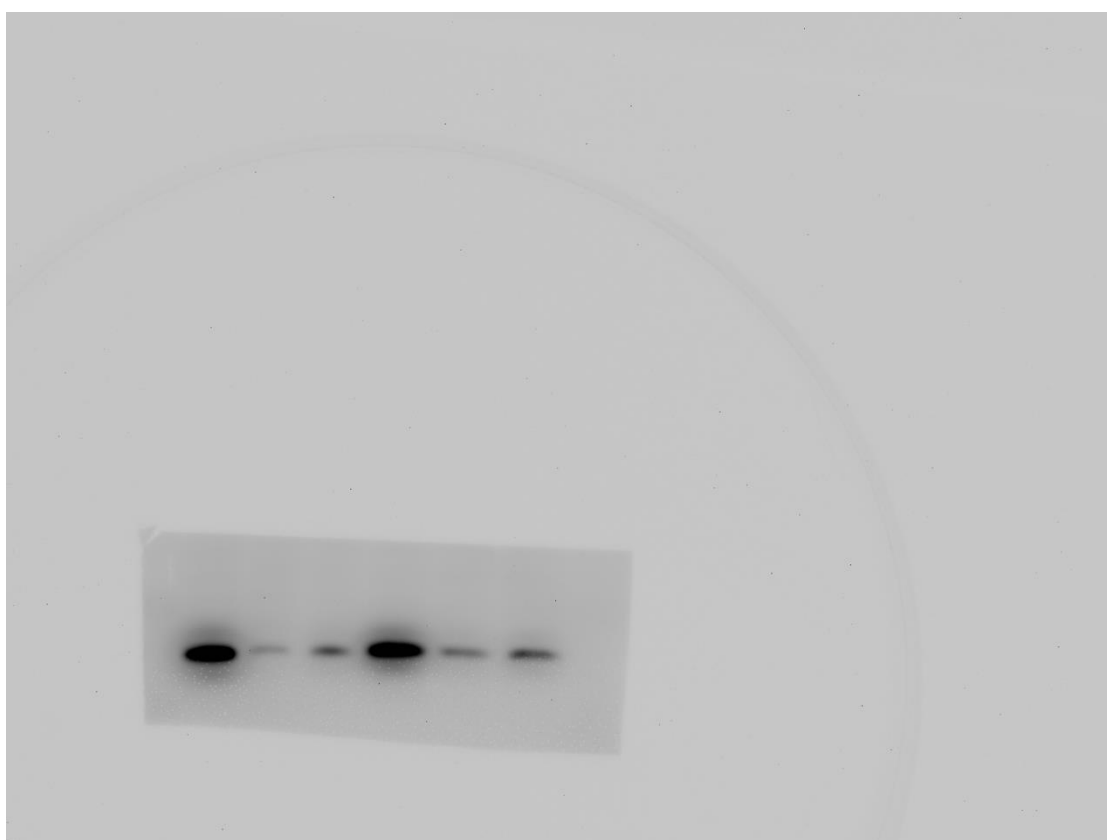

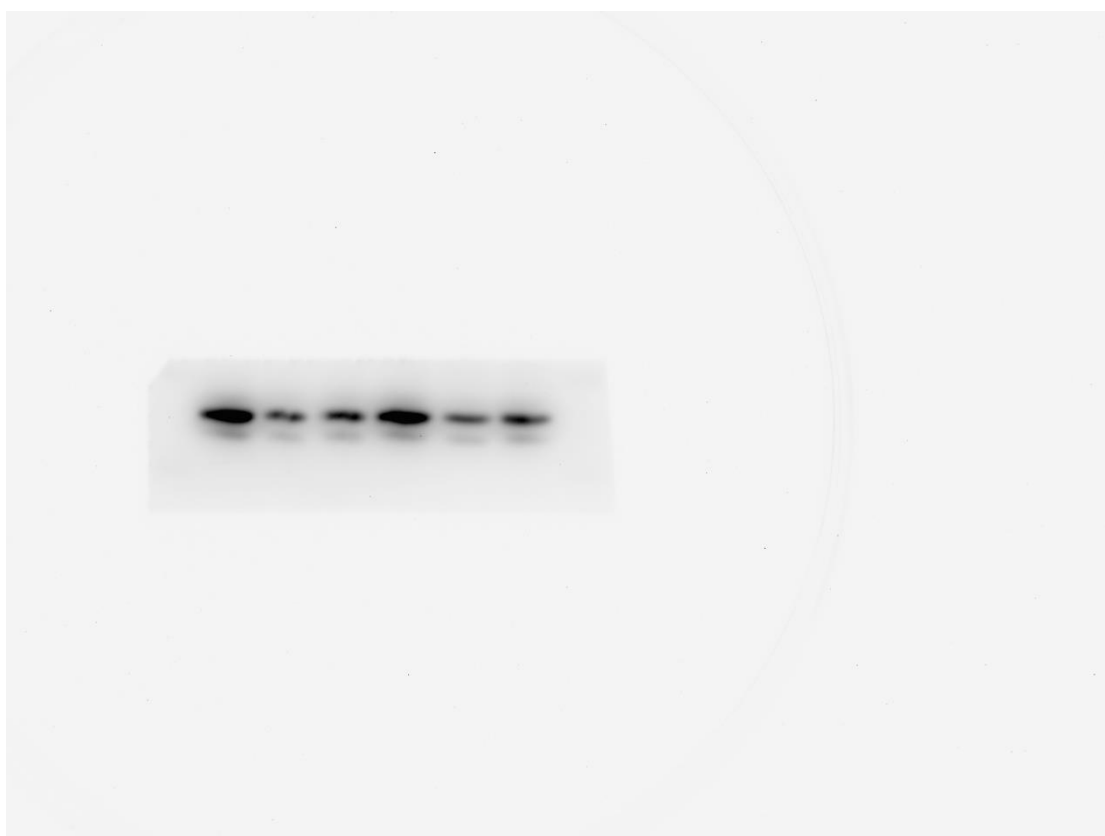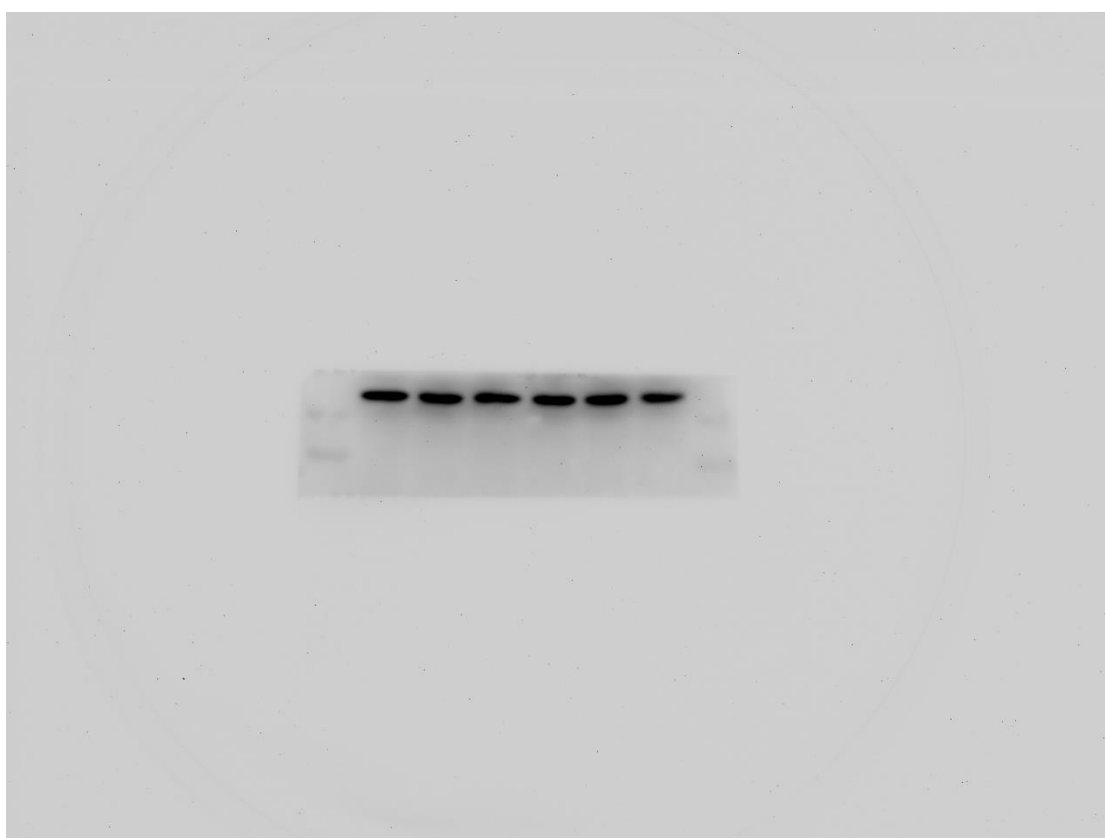

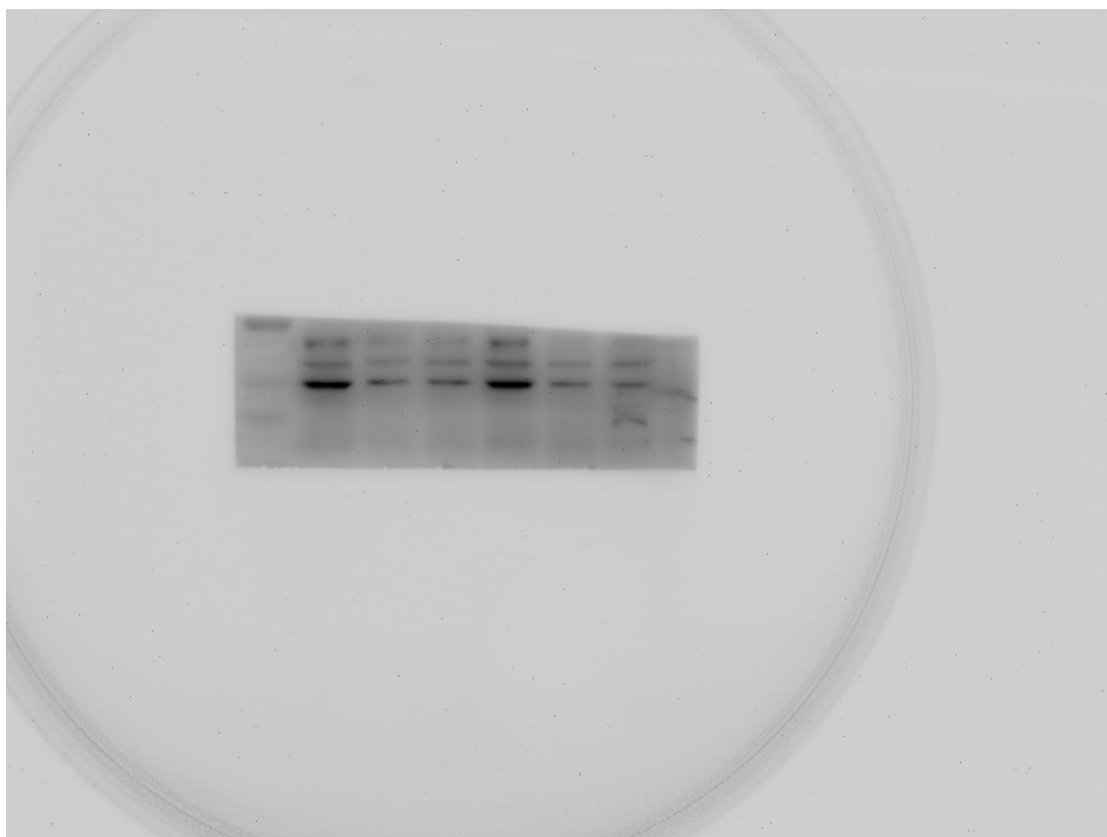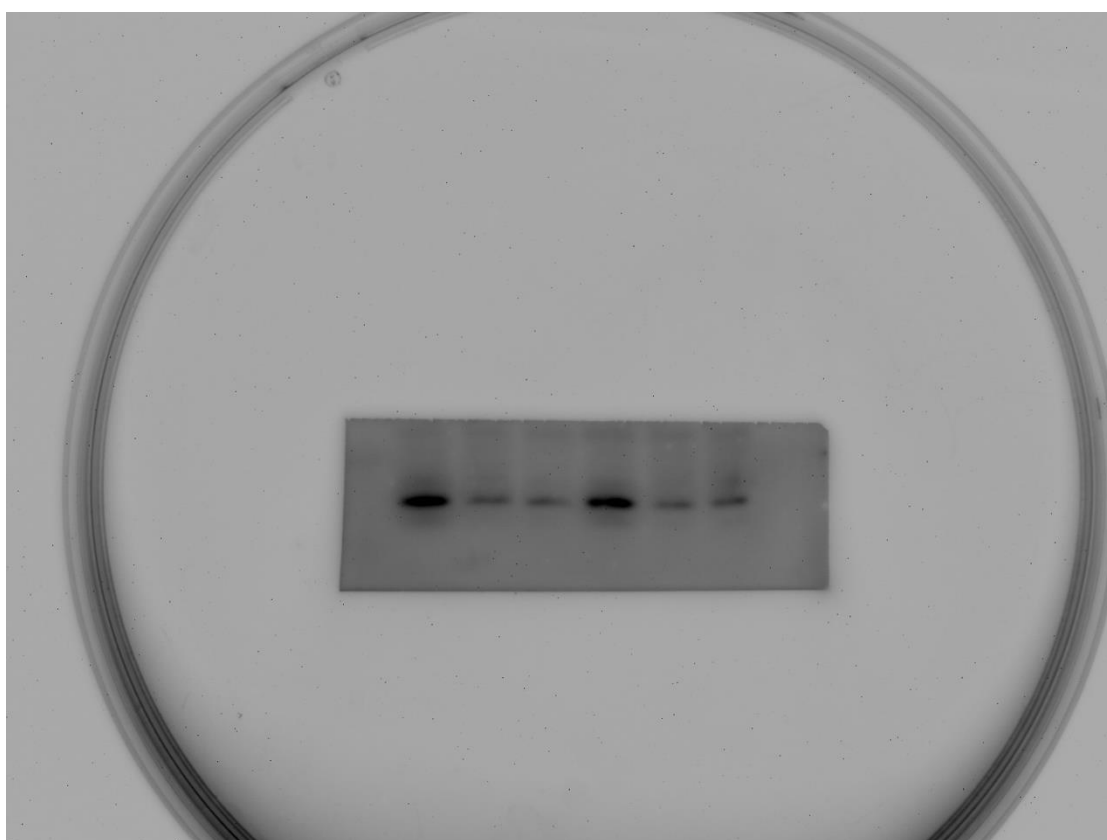

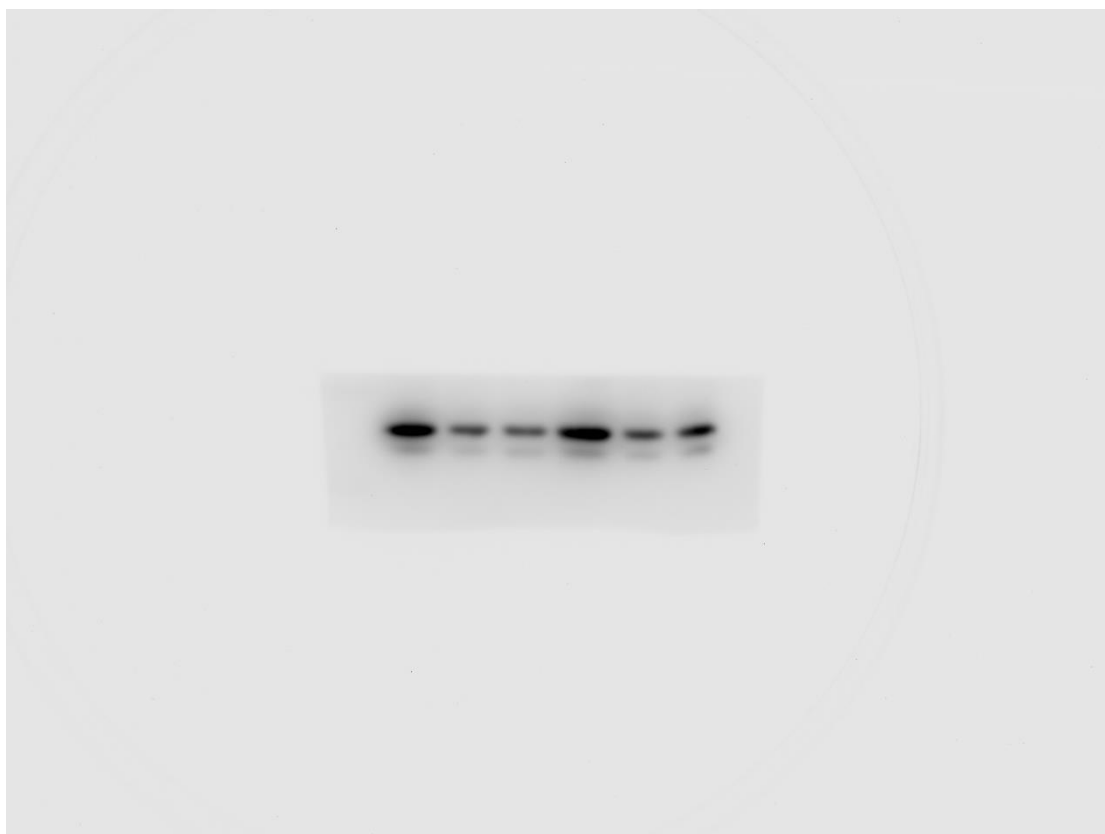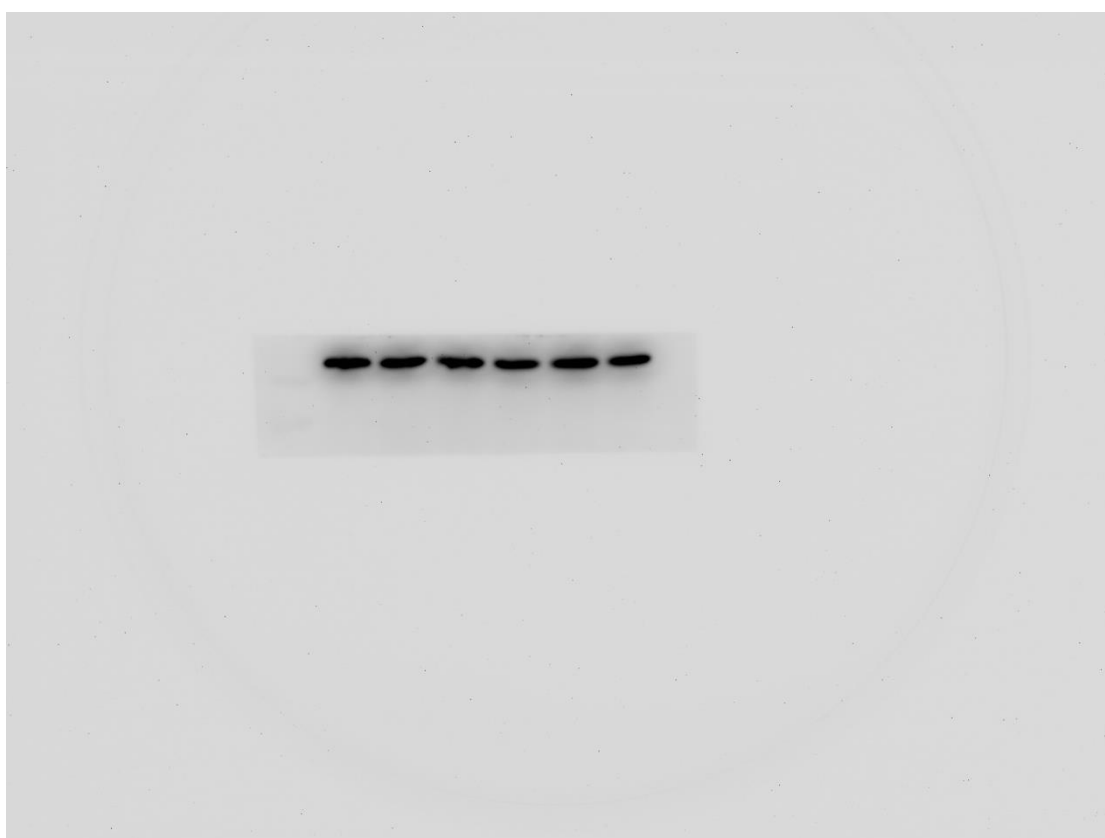

Supplement: Supplementary file 10 — Additional File 10: Original Western Blots. [file 13578_2023_1054_MOESM10_ESM.pdf]
